# Sex Differences in Blood Pressure and Cardiovascular Disease in the UK Biobank: A Prospective Cohort Study

**DOI:** 10.1093/ajh/hpaf214

**Published:** 2025-11-04

**Authors:** Rebecca K Kelly, Katie Harris, Cheryl Carcel, Paul Muntner, Mark Woodward

**Affiliations:** The George Institute for Global Health, University of New South Wales, Sydney, New South Wales, Australia; School of Medicine, College of Health and Medicine, University of Tasmania, Hobart, Tasmania, Australia; The George Institute for Global Health, University of New South Wales, Sydney, New South Wales, Australia; The George Institute for Global Health, University of New South Wales, Sydney, New South Wales, Australia; Department of Neurology, Royal Prince Alfred Hospital, Sydney, New South Wales, Australia; Department of Epidemiology, University of Alabama at Birmingham, Birmingham, AL, United States; The George Institute for Global Health, University of New South Wales, Sydney, New South Wales, Australia; The George Institute for Global Health, Imperial College London, London, United Kingdom

**Keywords:** sex differences, hypertension, coronary heart disease, stroke, blood pressure

## Abstract

**Background:**

Recent studies show that cardiovascular disease (CVD) risk increases from a lower nadir of systolic blood pressure (SBP) in women than men, and increases thereafter at a greater rate. This has led to a suggestion that sex-based SBP thresholds are required. We investigated sex differences in the associations of SBP and incident atherosclerotic CVD.

**Methods:**

This prospective study included 420,649 UK Biobank participants with no prior history of CVD. Age-adjusted sex-specific risks, relative risks (RRs), and risk differences (RDs) relating SBP to CVD were estimated using Poisson and Cox regression.

**Results:**

Over 13.6 years of follow-up, there were 28,628 CVD events. CVD risks across BP levels showed a “J-shape,” and were higher in men than women at all BP levels. The lowest risks were at SBP 100-<105 mmHg (events per 10,000 person-years [95% CI]: 15.6 [11.8-19.4]) and 110-<115 (47.2 [41.4-53.0]) among women and men, respectively. Compared with SBP 100-<110, sex-specific RRs at above 120 were higher in women than men, but RDs were higher in men than women at all levels of SBP. Compared to men at 110-<115 (ie, the men with least risk), risks in women were lower at all levels of SBP below 170.

**Conclusions:**

CVD risk is lowest for women at a slightly lower SBP than men and RRs for CVD increase with SBP at a slightly steeper rate in women. However, both risks and RDs in women are never greater than in men. This evidence does not support lower thresholds for diagnosis of hypertension in women.

## Graphical abstract

**Graphical Abstract:**
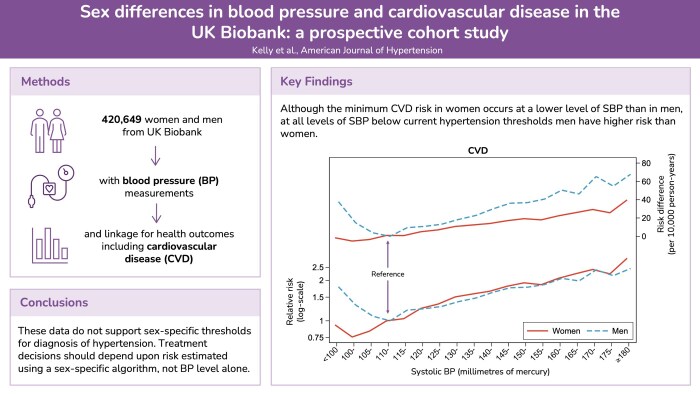

## Introduction

Hypertension guidelines do not currently provide sex-based recommendations for diagnosis or initiating blood pressure (BP)-lowering therapy, citing limited evidence.^1-3^ However, a recent scientific statement from the European Society of Cardiology acknowledges that sex-specific thresholds for hypertension diagnosis may be reasonable because the risk for cardiovascular disease (CVD) associated with higher BP starts at lower levels in women than men.^3^

Several observational studies have found that the relationship between BP and CVD differs by sex.^4-8^ These studies found higher relative risks (RRs) of CVD, compared to a low BP reference level, for women than men, as well as a lower nadir in the “J-shaped” association between BP and CVD in women than men.^4^^,^^8^ This has raised the question as to whether women should routinely be treated with antihypertensive medication at lower BP levels than men.^3^^,^^9^ However, none of these studies has reported this in the context of sex-specific risk differences (RDs), rather than RRs, or compared the RRs for women and men against a common reference group. In addition, prior meta-analyses have not found substantial sex difference in RRs for the relationship of BP with CVD events,^10–12^ or in the efficacy of BP-lowering treatments.^13^ Further research is needed to understand the potential impact of sex-specific thresholds for hypertension diagnosis on CVD outcomes.

To address these uncertainties, we sought to use data from a large cohort of data, with more CVD events than has been previously analyzed, enabling precise measures within smaller ranges of BP than have previously been used. Furthermore, we examine whether the sex-differential association between BP and CVD differs by age, and the effect menopause has on the BP-CVD relationship in women.^14^

## Methods

### Subjects and study design

The UK Biobank (UKB) is a prospective cohort study of approximately 500,000 men and women recruited between 2006 and 2010.^15^ Eligible adults aged between 40 and 69 years were invited to visit one of 22 centers for the baseline assessment, where detailed information on lifestyle and medical history was collected, as well as physical and biological measurements. Participants have been followed up for health-related outcomes via electronic health record linkage. Further details are available online.^15^ All individuals provided informed consent to participate, and the UKB study was approved by the National Information Governance Board for Health and Social Care and the National Health Service North West Multicentre Research Ethics Committee (reference number 21/NW/0157).

Participants were excluded from the present study if they withdrew consent (*n* = 961), or had a CVD history (self-reported or via linked electronic health records) prior to the baseline assessment (*n* = 40,270), or did not have two automated measurements of systolic BP (SBP) and diastolic BP (DBP) at baseline after excluding extreme measurements (*n* = 41,437) (Figure S1).

### Measurement of blood pressure

SBP and DBP, in millimeters of mercury, were calculated as the mean of two measurements taken at least 1 min apart at baseline, using an Omron HEM-7015IT digital BP monitor (Omron Healthcare) (see Supplementary Materials).^16^ Hypertension was defined as SBP ≥130 mmHg or DBP ≥80 mmHg, or current antihypertensive medication use, based on the American Heart Association (AHA) 2017 guidelines.^2^

### Outcomes

The primary outcome was incident atherosclerotic CVD, defined as a primary diagnosis of fatal or non-fatal coronary heart disease (CHD) or stroke. Secondary outcomes were incident CHD and stroke. The analyses were censored separately for incident CVD outcomes at the date of diagnosis or death, loss to follow-up, or administrative censoring (October 31, 2022 for England, August 31, 2022 for Scotland, May 31, 2022 for Wales) whichever occurred first. CHD was defined by a primary diagnosis of incident CHD (ICD-10 codes I21-I25) or coronary revascularization (OPCS-4 codes K49-K50, K75, K40-K46). Stroke was defined by a primary diagnosis of incident stroke (ICD-10 codes I60-I61, I63-I64).

### Statistical analyses

Baseline characteristics overall and by sex were presented as number (percentage) for categorical variables, or mean (SD) or median (interquartile interval) for continuous variables as appropriate.

Sex differences in the relationship of BP with CVD outcomes were explored using three approaches: (1) sex-specific risks and RDs, (2) sex-specific RRs, and (3) sex-combined RRs. Poisson regression models were used to obtain age-adjusted rates and rate differences of incident CVD events (per 10,000 person-years), by sex and BP group.^17^ Cox proportional hazards regression models were used to estimate hazard ratios for CVD.^17^ For simplicity and ease of interpretation, Poisson-derived rates and Cox-derived hazards ratios are hereafter referred to as “risks” and “RRs,” respectively. SBP and DBP were modelled using ordinal groups of 5 mmHg, with reference groups for BP chosen based on previous literature.^4^^,^^8^ For sex-specific analyses, SBP 110-<115 mmHg and DBP 70-<75 mmHg in women and men were taken as the reference groups for each sex separately. For sex-combined analyses, men with SBP 110-<115 mmHg and men with DBP 70-<75 mmHg were taken as the common reference groups for both sexes. Then the sex-specific BP-CVD association was modelled continuously in increments of 10 mmHg for SBP and 5 mmHg for DBP, for both sexes. Finally, BP was modelled using the 2017 ACC/AHA BP categories.^2^

All analyses were adjusted for age at recruitment, and multivariable models were additionally adjusted for the Townsend deprivation index, smoking status, body mass index (BMI), diabetes, antihypertensive and lipid-lowering medication use, total cholesterol, and high-density lipoprotein cholesterol (HDL-C). See Supplementary Materials for further details on covariate measurement. Percentages of missing for each covariate were <7%, except for HDL-C (14.0%) (Table S1).

#### Subgroup analyses

To investigate whether sex differences in the associations of BP with CVD differed by age group (<50, 50-<55, 55-<60, and ≥60 years), we added a three-way interaction between sex, age group, and the BP exposure of interest to the models. To assess trend across age groups separately for men and women (P-trend) and in the three-way interaction (P-interaction) we used likelihood ratio tests. Similarly, subgroup analyses were conducted by menopausal status in women (see Supplementary Materials).

#### Sensitivity analyses

We conducted sensitivity analyses to test the robustness of our findings by (1) excluding participants taking antihypertensive medication at baseline; (2) excluding participants with <2 years of follow-up, and (3) imputing missing covariate data via Multivariate Imputation by Chained Equations (MICE) over five iterations. To assess the potential influence of regression dilution bias we calculated sex-specific regression dilution ratios in a subsample of participants with BP measurements taken at the follow-up imaging assessment (2014+), on average, 10.6 years after the baseline assessment (*n* = 51,375, 8.2% of the main study sample).^18^ RRs were corrected for regression dilution bias by multiplying the regression coefficients by the reciprocal of the regression dilution ratio.

STATA version 18.0 (StataCorp LP, College Station, Texas) was used for data analyses and R 4.3.3 (R Core Team, Vienna, Austria) was used to create figures.

## Results

The baseline characteristics of 420,649 participants (56.0% women) without prior CVD included in the present analyses are shown in Table 1. Among both women and men, participants with hypertension were older and had higher BMI, and a higher proportion were taking antihypertensive or lipid-lowering medication (Table S2). See Tables S3 and S4 for baseline characteristics stratified by sex and SBP categories.

**Table 1.** Baseline characteristics and incident cardiovascular disease events for 420,649 UK Biobank participants.

Characteristics	Overall	Women	Men
	(*n* = 420,649)	(*n* = 235,556)	(*n* = 185,093)
**Incident total CVD cases during follow-up, *n***	28,628	10,331	18,297
**Incident CHD cases during follow-up, *n***	21,146	6,774	14,372
**Incident stroke cases during follow-up, *n***	8,321	3,816	4,505
**Age, mean (SD)**	56.1 (8.1)	56.1 (8.0)	56.1 (8.2)
**Ethnicity**			
** White**	395,023 (94.4%)	221,497 (94.4%)	173,526 (94.3%)
** Other** ^a^	23,587 (5.6%)	13,096 (5.6%)	10,491 (5.7%)
**Socioeconomic status**			
** Townsend deprivation index score, median (IQR)**	-2.2 (-3.7, −0.4)	-2.2 (-3.6, −0.4)	-2.2 (-3.7, −0.5)
** Townsend deprivation fifths:**			
** First (least deprived)**	158,076 (37.6%)	88,349 (37.5%)	69,727 (37.7%)
** Second**	86,275 (20.5%)	48,820 (20.7%)	37,455 (20.2%)
** Third**	62,888 (15.0%)	35,753 (15.2%)	27,135 (14.7%)
** Fourth**	56,033 (13.3%)	31,621 (13.4%)	24,412 (13.2%)
** Fifth (most deprived)**	57,377 (13.6%)	31,013 (13.2%)	26,364 (14.2%)
**Smoking status:**			
** Never**	234,198 (56.0%)	141,014 (60.2%)	93,184 (50.6%)
** Previous**	141,099 (33.7%)	72,978 (31.1%)	68,121 (37.0%)
** Current light (<15 cigarettes/day)**	13,015 (3.1%)	7,698 (3.3%)	5,317 (2.9%)
** Current medium (15-<30 cigarettes/day)**	14,309 (3.4%)	7,061 (3.0%)	7,248 (3.9%)
** Current heavy (>30 cigarettes/day)**	15,966 (3.8%)	5,676 (2.4%)	10,290 (5.6%)
**Diabetes**	47,783 (11.4%)	24,742 (10.5%)	23,041 (12.5%)
**Medication use:**			
** Antihypertensive medication use**	95,299 (22.7%)	47,265 (20.1%)	48,034 (26.0%)
** Lipid-lowering medication use**	56,537 (13.4%)	24,759 (10.5%)	31,778 (17.2%)
**Blood pressure (mmHg):**			
** SBP, mean (SD)**	137.7 (18.6)	135.1 (19.2)	141.0 (17.3)
** DBP, mean (SD)**	82.4 (10.1)	80.7 (9.9)	84.4 (9.9)
** Hypertension**	304,617 (72.4%)	155,855 (66.2%)	148,762 (80.4%)
** BMI (kg/m^2^):**			
** BMI, mean (SD)**	27.3 (4.7)	26.9 (5.1)	27.7 (4.2)
** Overweight (25-<30)**	178,943 (42.5%)	86,903 (36.9%)	92,040 (49.7%)
** Obesity (≥30)**	98,752 (23.5%)	53,836 (22.9%)	44,916 (24.3%)
**Serum lipids (mmol/L):**			
** Total cholesterol, mean (SD)**	5.8 (1.1)	5.9 (1.1)	5.6 (1.1)
** HDL-C, mean (SD)**	1.5 (0.4)	1.6 (0.4)	1.3 (0.3)

Numbers are participant numbers (%), with % representing the column percentage estimated excluding participants with missing responses, unless otherwise specified.

Abbreviations: BMI, body mass index; CHD, coronary heart disease; CVD, cardiovascular disease; DBP, diastolic blood pressure; HDL-C, high-density lipoprotein cholesterol; IQR, interquartile range; kg/m^2^, kilogram per square meter; mmHg, millimeters of mercury; mmol/L, millimoles per liter; SBP, systolic blood pressure.

a Includes Asian or Asian British, black or black British, Caribbean, African, any other black background, Chinese, other ethnic group, white and black Caribbean, white and black African, white and Asian, any other mixed background, Indian, Pakistani, Bangladeshi, any other Asian background.

### Sex-specific risks and RDs

**Figure 1.**
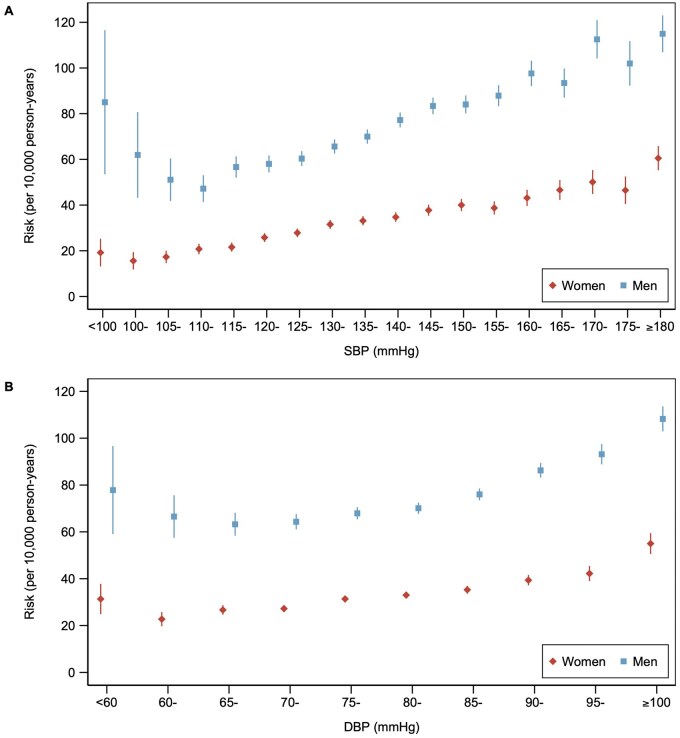
Risks of cardiovascular disease by level of blood pressure for each sex. Scatterplot showing age-adjusted risks (per 10,000 person-years) for incident cardiovascular disease across categories of (A) SBP and (B) DBP in women and men. Vertical lines indicate corresponding 95% confidence intervals. Abbreviations: DBP, diastolic blood pressure; mmHg, millimeters of mercury; SBP, systolic blood pressure.

During a median follow-up of 13.6 years, there were 28,628 (10,331 women), 21,146 (6,774 women), and 8,321 (3,816 women) cases of incident CVD, CHD, and stroke, respectively. Age-adjusted risks of incident CVD showed a “J-shaped” pattern and were higher among men in all categories of SBP and DBP (Figure 1). Risks of incident CVD were lowest at SBP 100-<105 mmHg for women (events per 10,000 person-years [95% CI], 15.6 [11.8-19.4]) and 110-<115 mmHg for men (47.2 [41.4-53.0]). Risks of incident CVD were lowest at DBP 60-<65 mmHg for women (22.7 [19.7-25.8]) and 65-<70 mmHg for men (63.3 [58.4-68.1]). Compared with SBP 100-<115 mmHg and DBP 70-<75 mmHg, sex-specific RDs at higher levels of BP were larger among men than women (Table S5). We found similar patterns for CHD and stroke (Figures S2 and S3 and Tables S6 and S7).

### Sex-specific RRs

**Figure 2.**
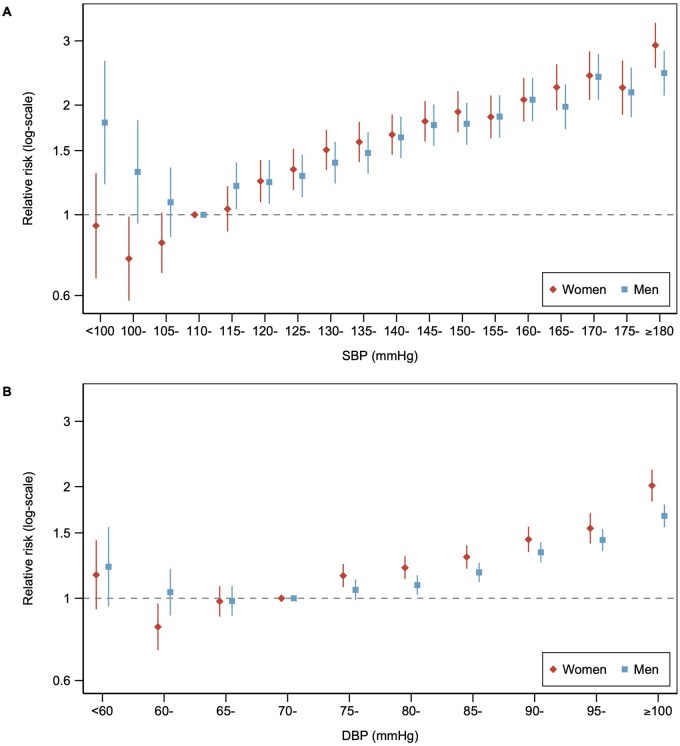
Sex-specific relative risks relating blood pressure to cardiovascular disease. Scatterplot showing age-adjusted sex-specific relative risks for incident cardiovascular disease across categories of (A) SBP and (B) DBP in women and men. Vertical lines indicate corresponding 95% confidence intervals. The reference category for SBP was 110-115 mmHg in both sexes separately. The reference category for DBP was 70-<75 mmHg in both sexes separately. Abbreviations: DBP, diastolic blood pressure; mmHg, millimeters of mercury; SBP, systolic blood pressure.

As would be expected from the risks, for SBP, there was an approximately “J-shaped” relationship for RRs of incident CVD for both sexes, which was roughly log-linear from nadirs of approximately 105 mmHg in women and 115 mmHg in men. Similarly, for DBP, with roughly log-linear increases from nadirs of approximately 65 mmHg in women and 75 mmHg in men (Figure 2). Sex-specific RRs for incident CVD, relative to 110-<115 mmHg for SBP and 70-<75 mmHg for DBP, were lowest at SBP 100-<105 mmHg (RR [95% CI], 0.76 [0.58-0.99]) and DBP 60-<65 mmHg (0.84 [0.72-0.97]) among women, and lowest at SBP 110-<115 (reference category) and DBP 65-<70 mmHg (0.98 [0.90-1.08]) for men, although CIs overlapped between adjacent BP groups. The association between higher SBP and increased RR was steeper among women compared with men. The patterns of sex differences in the associations of BP categories in relation to incident CHD and stroke were broadly similar, but with expected wider CIs (Figures S4 and S5). The association between hypertension and CVD was stronger in women (RR hypertension vs no hypertension [95% CI], 1.62 [1.54-1.70]) than men (1.45 [1.39-1.52]) (Table S8). Stronger RRs for the associations between continuous BP and CVD were present among women than men. Equivalent, broadly similar, values for multivariable-adjusted RRs for the sex-specific associations of BP with all CVD outcomes are shown in Tables S8-S10.

### Sex-combined RRs

**Figure 3.**
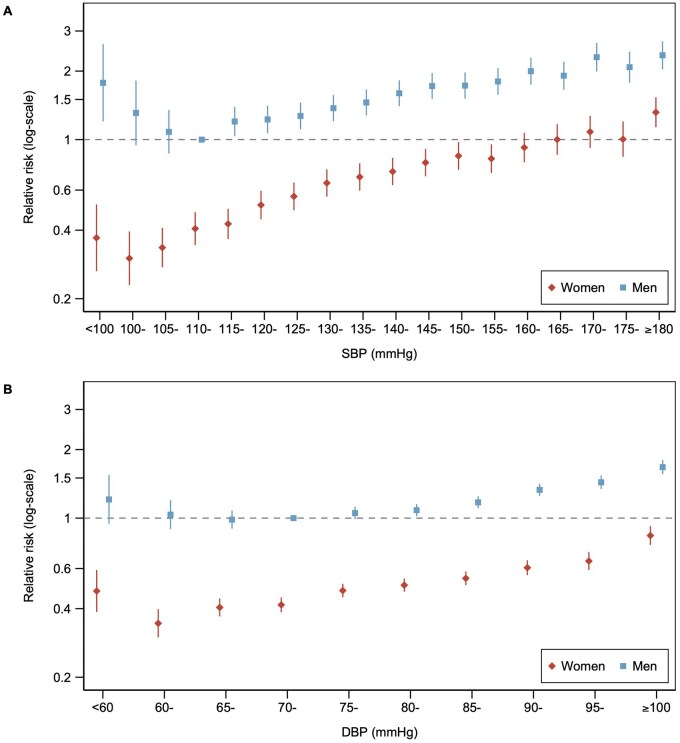
Sex-combined relative risks relating blood pressure to cardiovascular disease. Scatterplot showing age-adjusted sex-specific relative risks for incident cardiovascular disease across categories of (A) SBP and (B) DBP in women and men. Vertical lines indicate corresponding 95% confidence intervals. The reference category for SBP was men at 110-115 mmHg for both women and men. The reference category for DBP was men at 70-<75 mmHg for both women and men. Abbreviations: DBP, diastolic blood pressure; mmHg, millimeters of mercury; SBP, systolic blood pressure.

When comparing both women and men to the same reference group, men with SBP 110-<115 mmHg (that is, men who had the lowest CVD risk), RRs of incident CVD in women were never higher than until up to the 170-<175 mmHg group and peaked at >180 mmHg when the RR was 1.32 (1.13, 1.53) (Figure 3). So, it is only women with SBP above 170 mmHg that had greater CVD risk than any man, in terms of risk according to SBP level. For DBP level, no category of women had higher risks than the lowest risk group for men of 70-<75 mmHg. Similar patterns were observed for CHD and stroke (Figures S6 and S7). See Tables S11-S13 for age-adjusted and multivariable-adjusted sex-combined RRs for all CVD outcomes.

### Subgroup analyses

**Figure 4.**
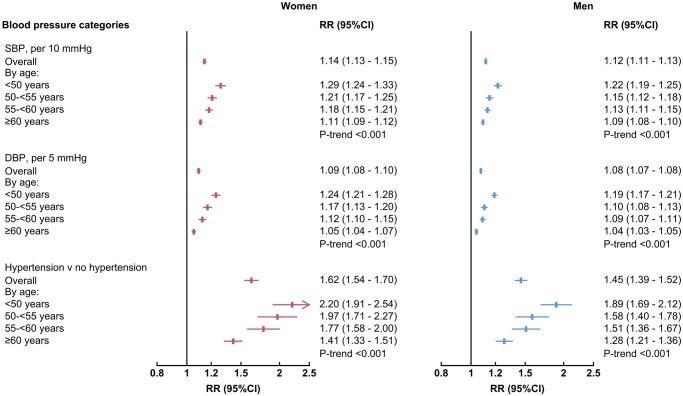
Sex-specific relative risks relating blood pressure level to cardiovascular disease by sex and age group. Forest plots showing sex and age-specific RRs relating continuous blood pressure and hypertension status to incident cardiovascular disease in women and men. Horizontal lines indicate corresponding 95% confidence intervals. Abbreviations: CI, confidence interval; DBP, diastolic blood pressure; mmHg, millimeters of mercury; SBP, systolic blood pressure; RR, relative risk.

The patterns of BP-CVD relationships were much the same in subgroup analyses by age and by menopausal status in women (Figure 4, Tables S14 and S15). RRs were greater for the associations between BP and incident CVD among younger age groups for both sexes and for pre-menopausal women compared with menopausal women, presumably due to the increasing risk with age.

### Sensitivity analyses

When excluding participants taking antihypertensives at baseline, the directions of association remained similar, although the magnitude of RRs for CVD generally became stronger (Table S16). Our findings remained similar when restricting to participants with ≥2 years of follow-up (Table S17) and after imputation for missing covariate data using MICE (Table S18).

Among 51,375 participants with baseline and follow-up BP measurements, regression dilution ratios for SBP and DBP were estimated to be 0.648 and 0.568, respectively, for women and 0.556 and 0.461, respectively, for men (Figures S8 and S9). Baseline characteristics were similar between participants with baseline and follow-up BP measurements and participants in the main study sample (Table S19). After correction for regression dilution, the sex-specific age-adjusted RRs by SBP and DBP levels for women moved slightly closer to those for men (Figure S10).

## Discussion

This large study assessed sex differences in the associations of BP with CVD, as well as CHD and stroke. CVD risks were higher among men than women at all levels of BP. There was a clear indication of a log-linear association starting substantially lower than current AHA treatment thresholds of SBP >130 mmHg and DBP >80 mmHg for both sexes. There was a “J-shaped” pattern relating increasing BP to CVD outcomes in both sexes, with a lower nadir in women (SBP 100-<105 mmHg and DBP 60-<65 mmHg) than men (SBP 110-<115 mmHg and DBP 65-<70 mmHg). RRs comparing to the reference groups of SBP 110-<115 or DBP 70-<75 were mostly higher for women than for men when the sexes were analyzed totally separately, but RDs showed the opposite relationship (Graphical Abstract). Furthermore, when a common reference group of men with SBP at 110-115 mmHg or men at DBP 70-<75 mmHg was used, when analyzing the sexes together, the RRs for women were lower than for men at all BP levels, whilst the risk of CVD in women only exceeded that of men at their lowest level of risk at high levels of BP.

There is established evidence that exceeding the existing thresholds for normal BP (SBP >120 mmHg or DBP >80 mmHg) is associated with an elevated risk of CVD in both sexes.^19^^,^^20^ However, few studies have explored associations of BP levels below these thresholds with CVD risks.^4^^,^^8^^,^^21^ Our results are broadly consistent with analyses from two US cohorts,^4^^,^^8^ reporting that CVD risk is lowest at lower SBP levels in women than men. For example, a study of 53,289 participants in the National Health and Nutrition Examination Survey (NHANES) found CVD mortality was lowest at SBP 100-<110 mmHg in women and 110-<120 mmHg in men.^8^ Other studies have considered CHD and stroke and, as in the current study, similar patterns were reported to those with CVD.^4^^,^^6^ Our study extends these findings by systematically considering both absolute risk and RR associations, considering narrower BP categories, jointly examining RRs and RDs, and comparing both sexes to a common reference group, providing a more nuanced interpretation of sex differences in BP-CVD relationships and a clearer basis for suitable clinical guidelines. We observed higher risks present at around SBP <100 mmHg and DBP <60 mmHg, compared to slightly higher BP levels, for both sexes. This appeared to be more pronounced for SBP among men, as well as among women who were menopausal or aged ≥55 years in subgroup analyses. One explanation is that lower SBP is associated with higher mortality among patients with heart failure^22^ and older adults (≥80) with frailty,^23^ although our study excluded those with pre-existing CVD and was predominantly middle-aged.

Prior epidemiological studies suggest that the strength of associations between BP and CVD varies by sex.^4^^,^^5^^,^^7^^,^^8^^,^^24^^,^^25^ Meta-analyses of observational studies have found that RRs for the association of BP with CVD events^12^ and mortality^11^^,^^26^ are stronger in women than men, although all studies concluded that these sex-based differences were not substantial. The most recent meta-analysis of 11 observational studies reported that AHA stage one hypertension was associated with 50% and 37% higher risks of CVD compared to normal BP in women and men, respectively.^12^ Similarly, our study found that hypertension was associated with a higher excess risk for incident CVD in women (46% higher) than men (32% higher) compared to no hypertension. However, CVD risks remained higher in men than women for hypertension and across all levels of BP.

Studies consistently demonstrate that women have lower mean basal BP than men.^27^^,^^28^ The trajectory of BP may differ by sex over time, with women exhibiting faster rise in BP from as early as the third decade of life.^29^ This study examined the roles of age and menopausal status, finding higher BP was associated with larger RRs for CVD among women that were pre-menopausal or aged <55 years. The steeper risk gradient observed in younger and pre-menopausal women suggests that initiating blood pressure screening prior to age 55, particularly around the average age of menopause and in women with reproductive or metabolic risk factors, may improve early detection and risk stratification.

Hormones, as well as anatomic and physiological differences, may explain sex differences in optimal BP and BP trajectories over time.^9^ Specifically, estrogen may exert beneficial effects on the renin-angiotensin-aldosterone system influencing BP homeostasis, and increased plasma renin and sympathetic nerve activity and decreased nitric oxide have been observed in post-menopausal women.^30–32^ Compared with men, women have smaller coronary arteries, smaller aortic root dimensions, and lower aortic distensibility with age, as well as higher pulse pressure, which may make women more susceptible to the cardiovascular effects of hypertension.^33–35^ Moreover, some conditions specific to women, including premature menopause and pregnancy-associated hypertension, may worsen CVD outcomes when coincident with hypertension.^3^^,^^36^ Lastly, emerging evidence suggests that the effect of genetic variants for BP is greater in women than men.^37^

Treatment for high BP is typically through antihypertensive drug therapy, although lifestyle changes, including salt reduction, can be expected to assist. In questioning whether women should be treated differently to men it is necessary to consider whether they react to the drugs differently. Individual clinical trials have described sex differences in the efficacy, pharmacodynamics, and pharmacokinetics, and overall, women report more adverse effects from antihypertensive medication.^3^^,^^9^ However, the BP Lowering Treatment Trialists’ Collaboration has found that, with antihypertensive drug treatment, BP reductions were similar for women and men in every comparison they made, and there was no sex difference in protection against CVD regardless of age or antihypertensive regimen.^13^^,^^26^ Our findings support current guidelines recommending risk-based treatment thresholds rather than sex-specific BP thresholds, as women’s RRs increase more steeply but absolute risks remain lower than men’s across all BP levels.^1–3^ RRs alone, including those presented in this study, do not necessarily translate into treatment benefit,^38^ and treatment thresholds should be informed by randomized trials with outcome data rather than risk estimates alone.

Strengths of this study include the prospective study design and large sample size, which allowed us to examine smaller ranges of BP than previously studied, as well as major CVD subtypes. We also considered the roles of age and menopausal status in more detail than prior studies. This study has several limitations. The main analyses used BP measurements taken at a single baseline visit, which may be prone to measurement error.^1^ Indeed, our assessment of regression dilution bias in BP using participants with repeat measurements suggests this may have led to an underestimation of reported RRs, particularly among men. Because of the extensive baseline data available in UKB, we were able to account for potential confounders, including serum total cholesterol and HDL-C. However, this study was unable to account for changes in these factors during the follow-up period, and we cannot exclude the effects of unmeasured and residual confounding, particularly in antihypertensive medication use. Reverse causation also cannot be ruled out, although we excluded participants with prior CVD at baseline. While a J-shaped pattern was observed for CVD risks across BP levels, these results should be interpreted cautiously because of the wide confidence intervals at lower BP levels. Finally, the UKB study population is not nationally representative, being predominantly of white ethnicity and higher socioeconomic status, so our findings may be prone to selection bias, particularly for risk estimation. However, we have no reason to suppose that such biases would affect the sexes differently, and thus that this is unlikely to influence sex differences which are the focus of this study. Lastly, we only studied CVD, whereas normal or optimal BP for other disease outcomes may differ, and the potential adverse effects at lower BP levels were not examined.

In this study, we have separated the effects of BP levels by risk and RR, which have been confused in past literature and considered narrow increments of BP.^39^ Our analyses suggest that the level at which hypertension is defined should not vary by sex. While treatment decisions should be guided by overall cardiovascular risk using sex-specific algorithms,^38^^,^^40^ thresholds for defining hypertension remain critical for screening, risk communication, and guideline formulation.

## Supplementary Material

hpaf214_Supplementary_Data

## Data Availability

This research has been conducted using the UK Biobank Resource under application number 74018. Bona fide researchers can apply to use the UK Biobank dataset by registering and applying at http://ukbiobank.ac.uk/register-apply/.
