# Supplementary material for: Sex Differences in Blood Pressure and Cardiovascular Disease in the UK Biobank: A Prospective Cohort Study
Source: Am J Hypertens. 2025 Nov 4;39(1):98–107. doi: 10.1093/ajh/hpaf214 (PMC12802948; doi:10.1093/ajh/hpaf214)
Supplement: hpaf214_Supplementary_Data [file hpaf214_supplementary_data.docx]

**SUPPLEMENTARY MATERIALS**

**Sex differences in blood pressure and cardiovascular disease in the UK Biobank: a prospective cohort study**

Rebecca K Kelly, MD, PhD, Katie Harris, PhD, Cheryl Carcel, MD, PhD, Paul Muntner, PhD, Mark Woodward, PhD

[SUPPLEMENTAL METHODS 4](#_Toc208573898)

[SUPPLEMENTAL FIGURES 6](#_Toc208573899)

[Figure S1. Flow chart of participants included in the current study. 6](#_Toc208573900)

[Figure S2. Risks for BP and CHD by sex. 7](#_Toc208573901)

[Figure S3. Risks for BP and stroke by sex. 8](#_Toc208573902)

[Figure S4. Sex-specific RRs for BP and CHD. 9](#_Toc208573903)

[Figure S5. Sex-specific RRs for BP and stroke. 10](#_Toc208573904)

[Figure S6. Sex-combined RRs for BP and CHD. 11](#_Toc208573905)

[Figure S7. Sex-combined RRs for BP and stroke. 12](#_Toc208573906)

[Figure S8. Mean SBP at baseline and follow-up in fifths defined by baseline SBP. 13](#_Toc208573907)

[Figure S9. Mean DBP at baseline and follow-up in fifths defined by baseline DBP. 14](#_Toc208573908)

[Figure S10. Sex-specific RRs for BP and CVD with correction for regression dilution. 15](#_Toc208573909)

[SUPPLEMENTAL TABLES 16](#_Toc208573910)

[Table S1. Missing data in the sample for the current study. 16](#_Toc208573911)

[Table S2. Baseline characteristics of 420,649 UK Biobank participants included in the main analyses stratified by AHA hypertension category. 17](#_Toc208573912)

[Table S3. Baseline characteristics of 233,556 women in UK Biobank in the main analyses stratified by SBP categories. 19](#_Toc208573913)

[Table S4. Baseline characteristics of 185,093 men in UK Biobank in the main analyses stratified by SBP categories. 21](#_Toc208573914)

[Table S5. Risks and sex-specific RDs for BP and CVD by sex. 23](#_Toc208573915)

[Table S6. Risks and sex-specific RDs for BP and CHD by sex. 24](#_Toc208573916)

[Table S7. Risks and sex-specific RDs for BP and stroke by sex. 25](#_Toc208573917)

[Table S8. Sex-specific RRs for BP and CVD. 26](#_Toc208573918)

[Table S9. Sex-specific RRs for BP and CHD. 28](#_Toc208573919)

[Table S10. Sex-specific RRs for BP and stroke. 30](#_Toc208573920)

[Table S11. Sex-combined RRs for BP and CVD. 32](#_Toc208573921)

[Table S12. Sex-combined RRs for BP and CHD. 34](#_Toc208573922)

[Table S13. Sex-combined RRs for BP and stroke. 36](#_Toc208573923)

[Table S14. Sex-specific RRs for BP and CVD by age group. 38](#_Toc208573924)

[Table S15. Sex-specific RRs for BP and CVD by menopausal status among women participants only. 40](#_Toc208573925)

[Table S16. Sex-specific RRs for BP and CVD in sensitivity analyses restricting to participants not taking antihypertensive medication at baseline. 41](#_Toc208573926)

[Table S17. Sex-specific RRs for BP and CVD in sensitivity analyses restricting to participants with **≥**2 years of follow-up. 42](#_Toc208573927)

[Table S18. Sex-specific RRs for BP and CVD in sensitivity analyses imputing missing covariate data via Multivariate Imputation by Chained Equations (MICE) over five iterations. 43](#_Toc208573928)

[Table S19. Baseline characteristics of 51,375 UK Biobank participants with BP measurements at baseline (2006-2010) and follow-up (2014+) who were included in the subsample for assessment of regression dilution bias. 44](#_Toc208573929)

# SUPPLEMENTAL METHODS

**Blood pressure**

Physical measures were collected for all participants at baseline by trained research staff.^1^ Blood pressure was measured by trained research staff using an Omron HEM-7015IT digital blood pressure monitor. All blood pressure measurements were taken from participants while seated and from the left brachial artery, where possible. Systolic blood pressure (SBP) and diastolic blood pressure (DBP) in millimetres of mercury (mmHg) were calculated from the mean of two measurements. Implausible of measurements of SBP (<70 mmHg or >270 mmHg) and DBP (<30 mmHg or >150 mmHg) were removed.

**Age at recruitment**

Age at recruitment was calculated using participants’ month and year of birth, which was verified or updated by participants. Day of birth was assigned nominally as the 15^th^ of the month for each participant.

**Townsend deprivation index**

Townsend deprivation index was calculated by assigning each participant a score corresponding to the output area from the preceding national census in which their postcode was located. Participants were categorised into fifths from least to most deprived using national cut-off points (<-2.938 [least deprived]; -2.938-<-1.531; -1.531-<0170; 0.170-<2.448; and ≥2.448 [most deprived]) ^2^. Participants who were missing this score were categorised as unknown.

**Smoking status**

Participants self-reported smoking status in the Touchscreen questionnaire was used to classify participants as never smokers, former smokers, current smokers, or unknown. Current smokers were further grouped according to the number of cigarettes they smoke on average each day: light smokers (<15 cigarettes/d); medium smokers (15-<30 cigarettes/d); heavy smokers (≥30 cigarettes/d); or smoker of unknown number of cigarettes.

**Body mass index**

Standing height in centimetres (cm) was measured without shoes using the SECA 240 device. Weight in kilograms (kg) was measured using the Tanita BC 418 body composition analyser. If participants were unable or declined to undergo body composition analysis, weight was measured using standard scales. Body mass index (BMI) was calculated as weight(kg)/height(m)^2^.

**Diabetes**

Participants were classified as having diabetes based on self-reported diagnosis in the Touchscreen questionnaire and/or self-reported insulin or other glucose-lowering medication use (i.e. sulfonylureas, meglitinides, biguanides, thiazolidinediones and alpha-glucosidase inhibitors) in the prescriptions and over-the-counter medications section of the computer-assisted personal interview (CAPI). Participants were categorised as unknown only if their answers to all both questions were unknown.

**Antihypertensive medication use**

Participants were classified based on self-reported antihypertensive medication use (e.g. calcium-channel blockers, angiotensin-receptor blockers) in the prescriptions and over-the-counter medications section of the CAPI.

**Lipid-modifying medication use**

Participants were classified based on self-reported lipid-lowering medication use (i.e. statins, bile acid sequestrants, nicotinic acid or fibrates, or selective cholesterol absorption inhibitors) in the prescriptions and over-the-counter medications section of the CAPI.

**Total cholesterol and high-density lipoprotein cholesterol**

Non-fasting blood samples were collected from participants by trained phlebotomists and used to measure total cholesterol (mmol/L) and high-density lipoprotein cholesterol (HDL-C, mmol/L).

**Menopausal status**

Menopausal status in women (pre-menopausal, post-menopausal or unknown) was based on responses to the question “Have you had your menopause (periods stopped)?” in the Touchscreen questionnaire. If participants responded with ‘not sure had hysterectomy’, ‘not sure other reason’ or ‘prefer not to answer’, information from other questions on bilateral oophorectomy, use of menopausal hormone therapy (MHT), and age at recruitment were used to further categorise participants.

# SUPPLEMENTAL FIGURES


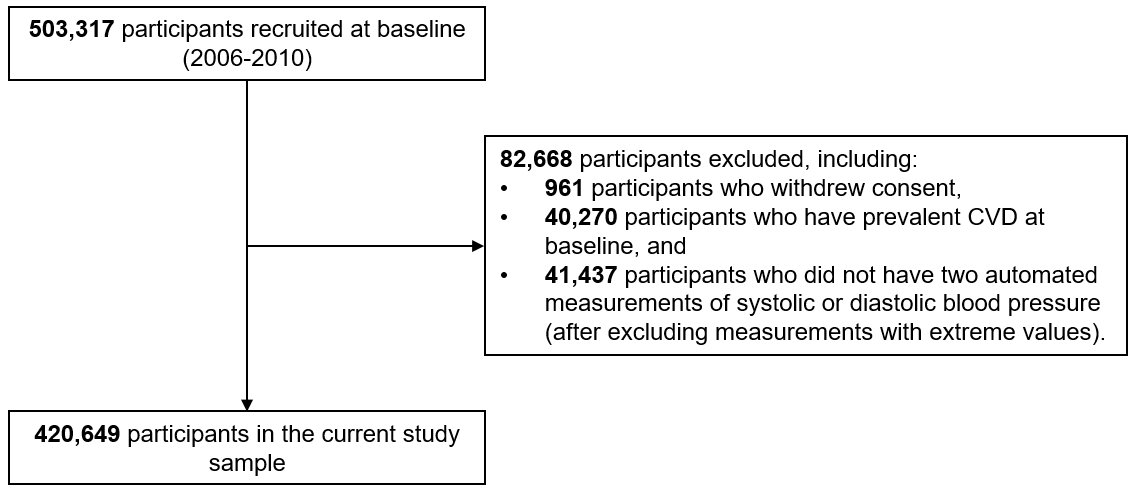


Figure S1. Flow chart of participants included in the current study.

Abbreviations: CVD, cardiovascular disease.


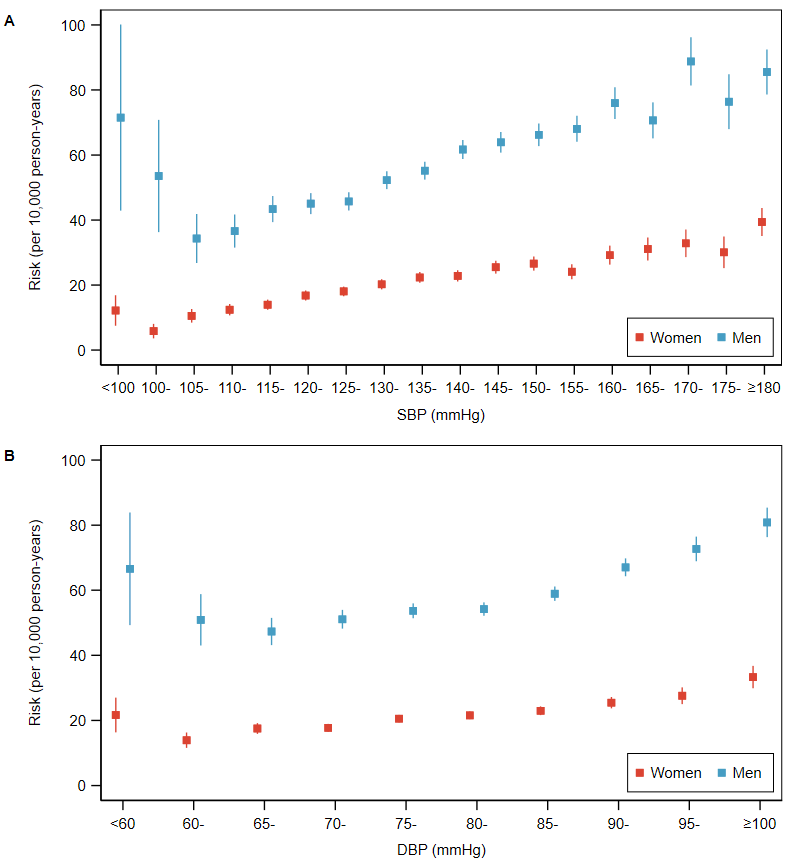


Figure S2. Risks for BP and CHD by sex.

Scatterplot showing age-adjusted risks (per 10,000 person-years) for incident CHD across categories of SBP (A) and DBP (B) in women (red) and men (blue). Horizontal lines indicate corresponding 95% confidence intervals around risks. Abbreviations: CHD, coronary heart disease; DBP, diastolic blood pressure; mmHg, millimetres of mercury; SBP, systolic blood pressure.


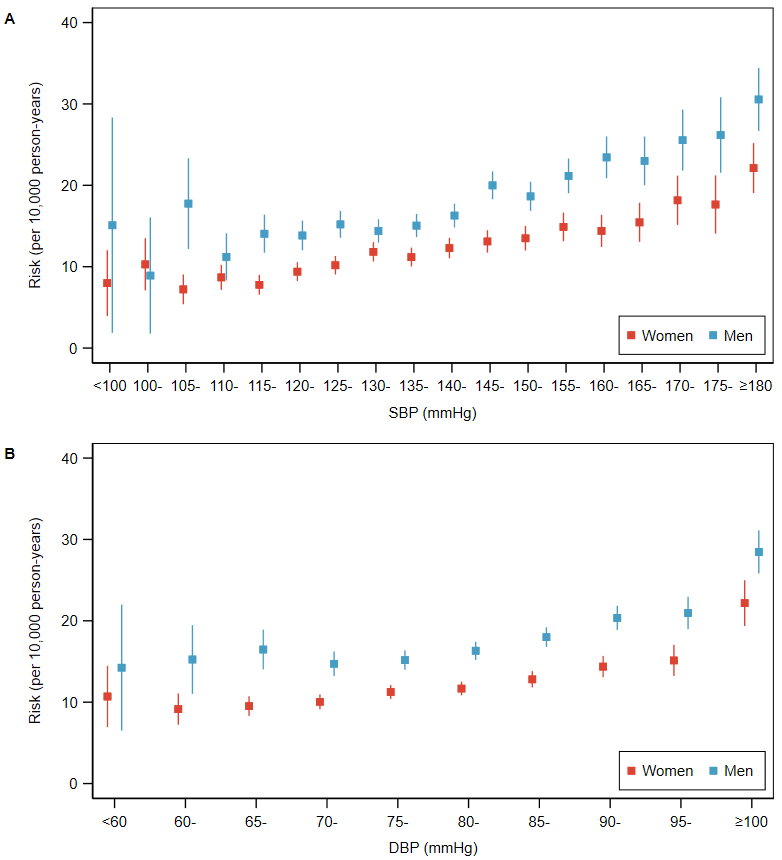


Figure S3. Risks for BP and stroke by sex.

Scatterplot showing age-adjusted risks (per 10,000 person-years) for incident stroke across categories of SBP (A) and DBP (B) in women (red) and men (blue). Horizontal lines indicate corresponding 95% confidence intervals around risks. Abbreviations: DBP, diastolic blood pressure; mmHg, millimetres of mercury; SBP, systolic blood pressure.


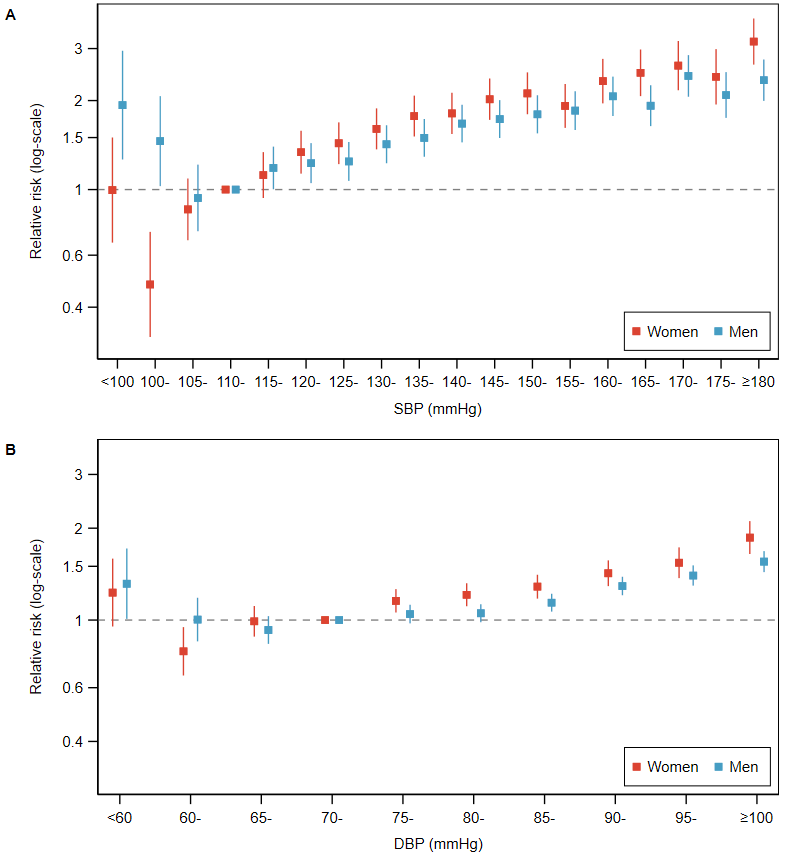


Figure S4. Sex-specific RRs for BP and CHD.

Scatterplot showing age-adjusted sex-specific RRs for incident CHD across categories of SBP (A) and DBP (B) in women (red) and men (blue). Horizontal lines indicate corresponding 95% confidence intervals around RRs. The reference category for SBP was 110-115 mmHg in both sexes separately. The reference category for DBP was 70-<75 mmHg in both sexes separately. Abbreviations: CHD, coronary heart disease; DBP, diastolic blood pressure; mmHg, millimetres of mercury; RR, relative risk; SBP, systolic blood pressure.


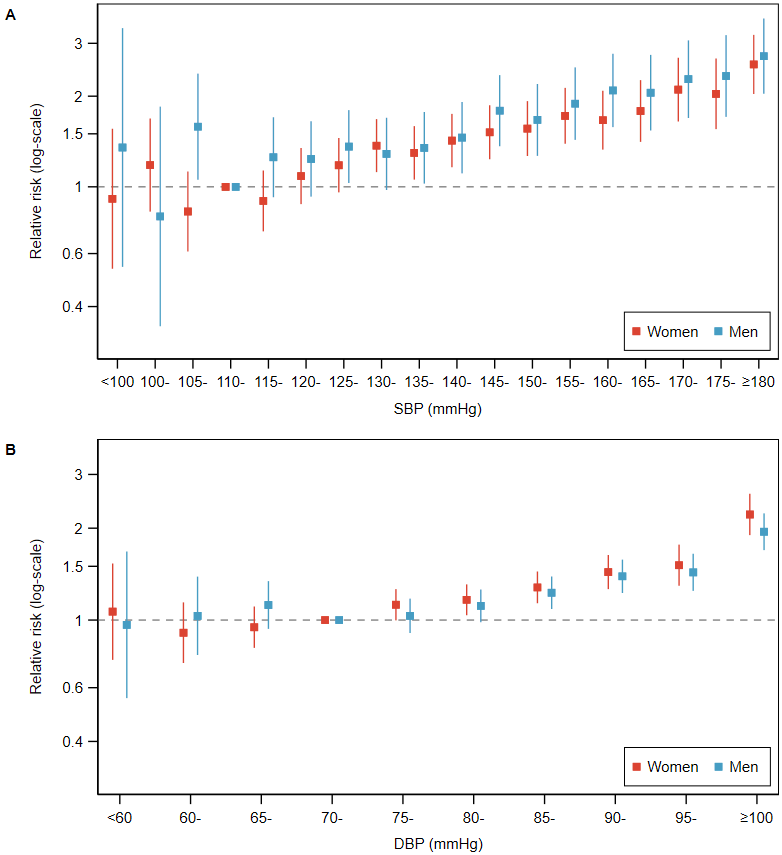


Figure S5. Sex-specific RRs for BP and stroke.

Scatterplot showing age-adjusted sex-specific RRs for incident stroke across categories of SBP (A) and DBP (B) in women (red) and men (blue). Horizontal lines indicate corresponding 95% confidence intervals around RRs. The reference category for SBP was 110-115 mmHg in both sexes separately. The reference category for DBP was 70-<75 mmHg in both sexes separately. Abbreviations: DBP, diastolic blood pressure; mmHg, millimetres of mercury; RR, relative risk; SBP, systolic blood pressure.


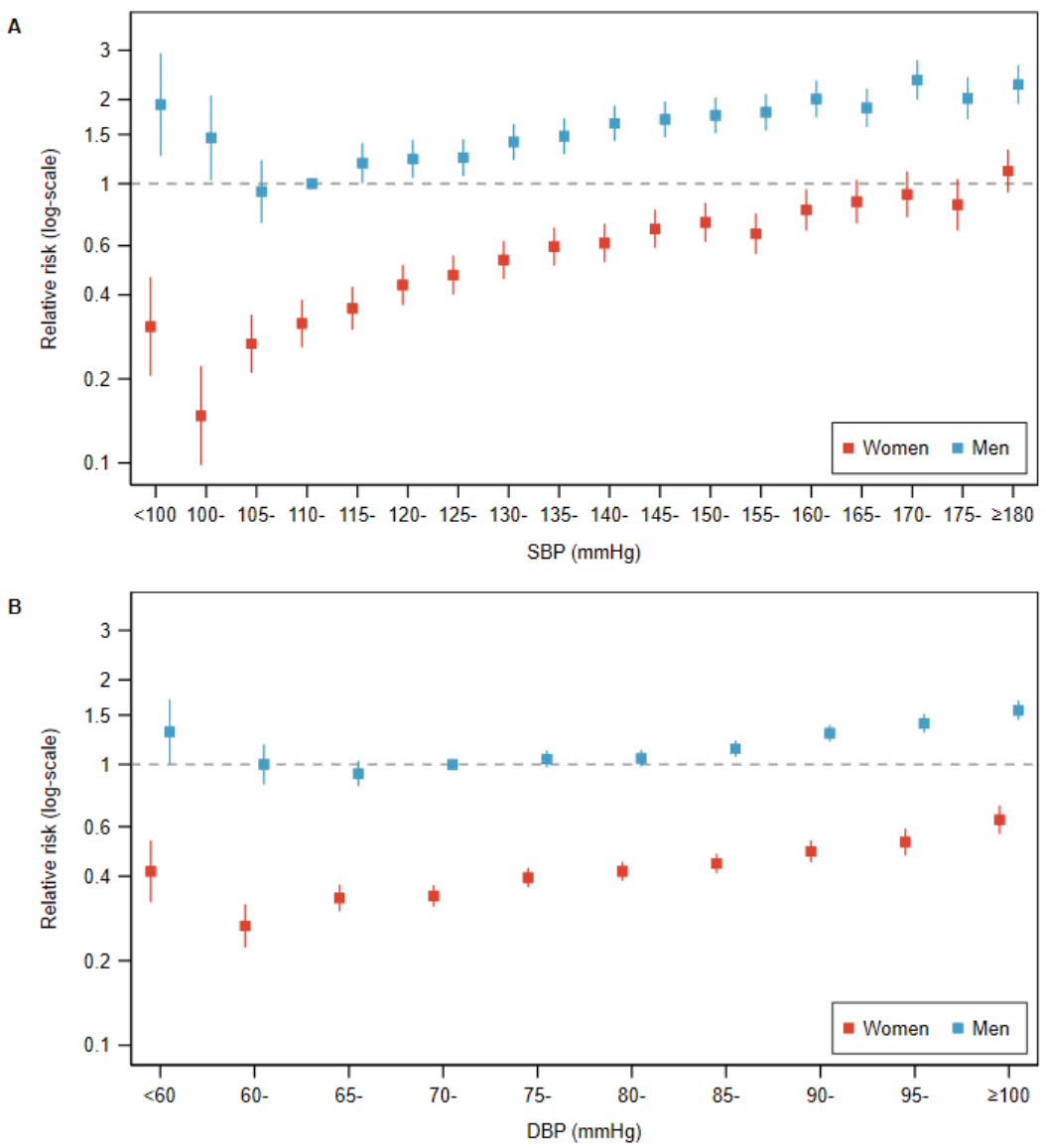


Figure S6. Sex-combined RRs for BP and CHD.

Scatterplot showing age-adjusted sex-combined RRs for incident CHD across categories of SBP (A) and DBP (B) in women (red) and men (blue). Horizontal lines indicate corresponding 95% confidence intervals around RRs. The reference category for SBP was 110-115 mmHg in men for women and men. The reference category for DBP was 70-<75 mmHg in men for women and men. Abbreviations: CHD, coronary heart disease; DBP, diastolic blood pressure; mmHg, millimetres of mercury; RR, relative risk; SBP, systolic blood pressure.


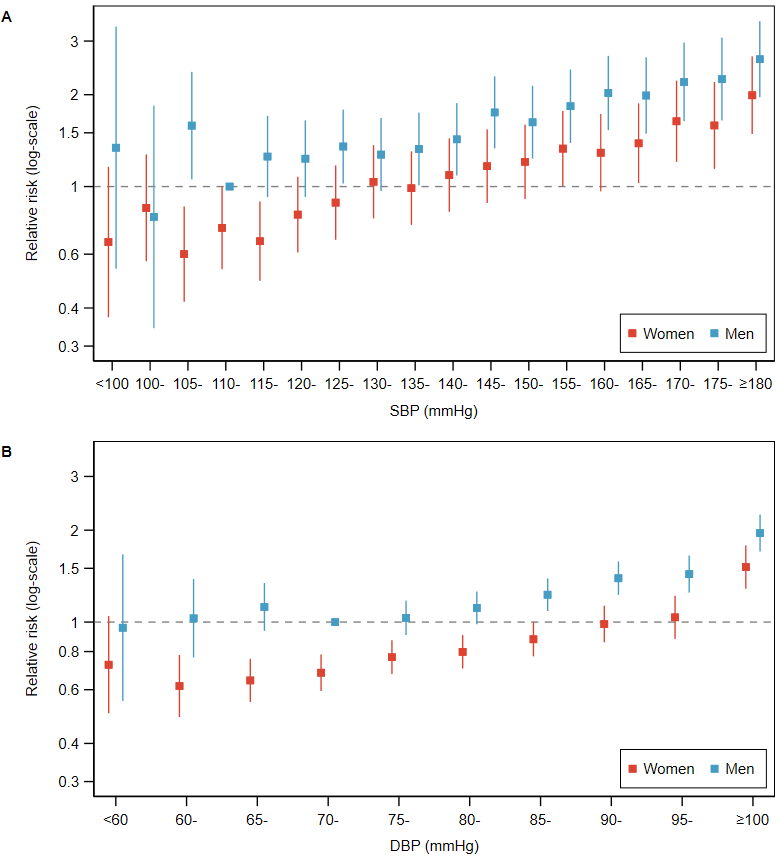


Figure S7. Sex-combined RRs for BP and stroke.

Scatterplot showing age-adjusted sex-combined RRs for incident stroke across categories of SBP (A) and DBP (B) in women (red) and men (blue). Horizontal lines indicate corresponding 95% confidence intervals around RRs. The reference category for SBP was 110-115 mmHg in men for women and men. The reference category for DBP was 70-<75 mmHg in men for women and men. Abbreviations: DBP, diastolic blood pressure; mmHg, millimetres of mercury; RR, relative risk; SBP, systolic blood pressure.

**
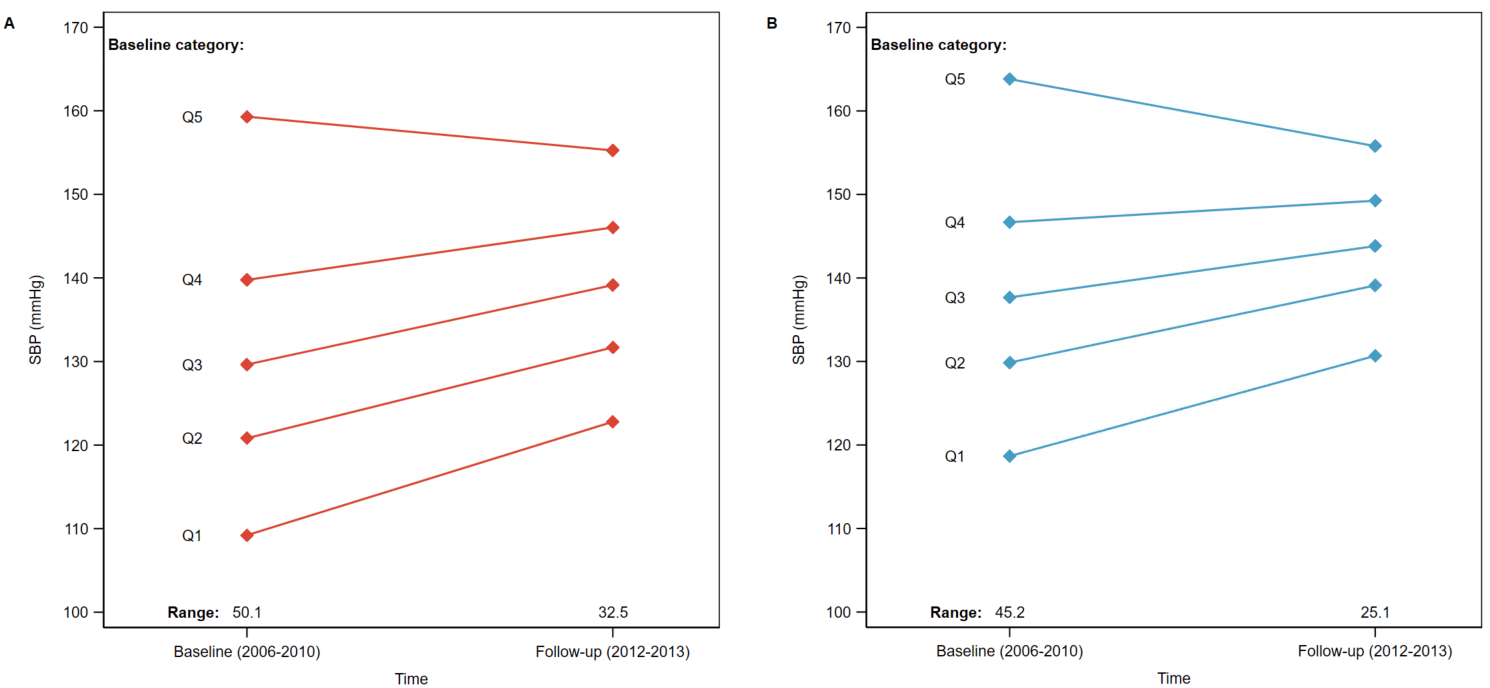
**

Figure S8. Mean SBP at baseline and follow-up in fifths defined by baseline SBP.

Mean SBP at baseline (2006-2010) and follow-up (2014+) in fifths defined by baseline SBP in women (A) and men (B) among 51,375 UK Biobank participants with baseline and follow-up SBP measurements. Range was calculated as the difference between the highest fifth (Q5) and lowest fifth (Q1) of baseline SBP. Regression dilution ratios were derived from the ratio of ranges between follow-up and baseline separately for women (regression dilution ratio = 0.648) and men (regression dilution ratio = 0.556) separately. Abbreviations: mmHg, millimetres of mercury; Q, quintile; SBP, systolic blood pressure.

**
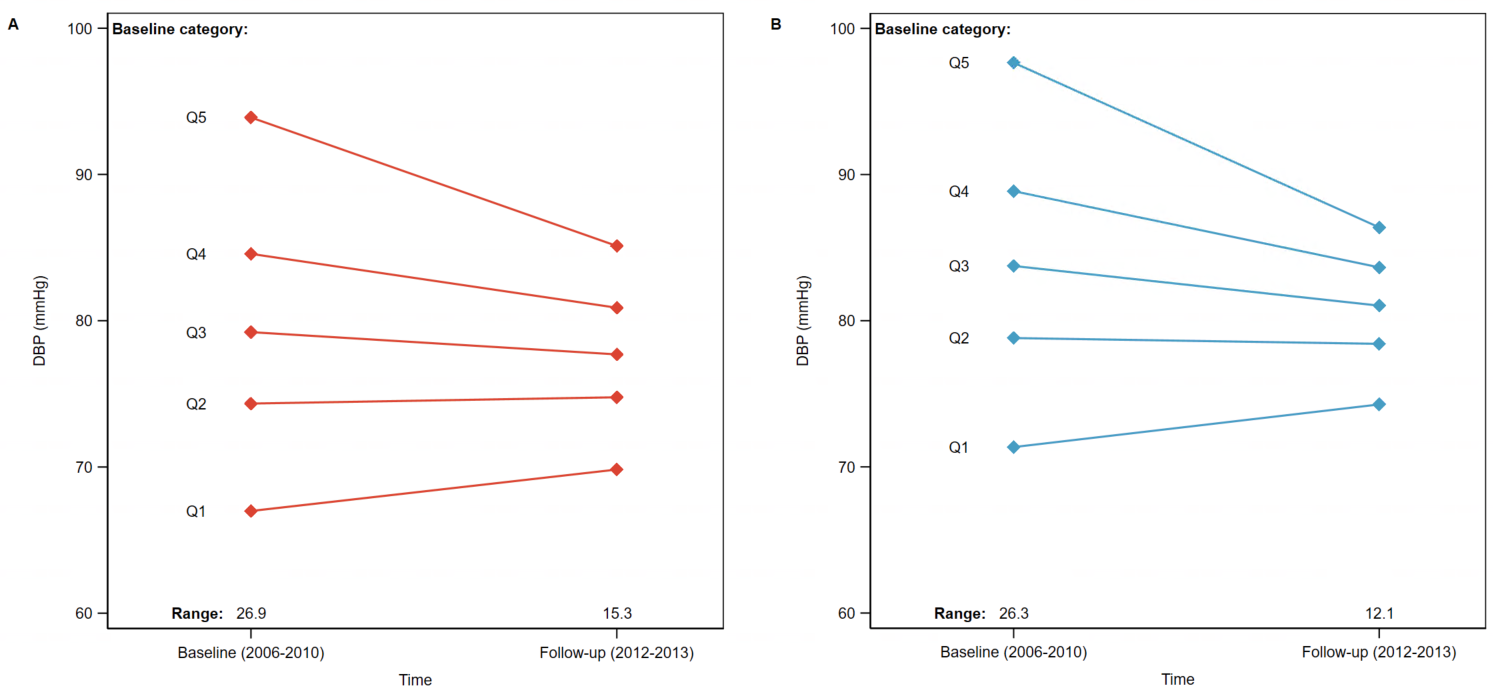
**

Figure S9. Mean DBP at baseline and follow-up in fifths defined by baseline DBP.

Mean DBP at baseline (2006-2010) and follow-up (2014+) in fifths defined by baseline DBP in women (A) and men (B) among 51,375 UK Biobank participants with baseline and follow-up DBP measurements. Range was calculated as the difference between the highest fifth (Q5) and lowest fifth (Q1) of baseline DBP. Regression dilution ratios were derived from the ratio of ranges between follow-up and baseline separately for women (regression dilution ratio = 0.568) and men (regression dilution ratio = 0.461) separately. Abbreviations: DBP, diastolic blood pressure; Q = quintile, mmHg, millimetres of mercury.


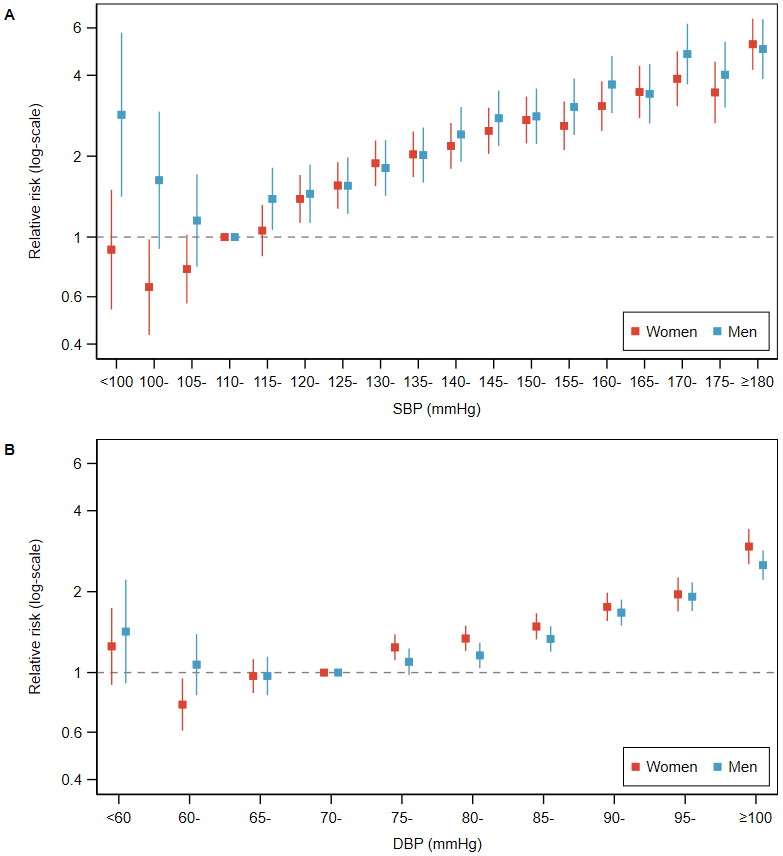


Figure S10. Sex-specific RRs for BP and CVD with correction for regression dilution.

Scatterplot showing age-adjusted sex-specific RRs for incident CVD across categories of SBP (A) and DBP (B) in women (red) and men (blue). Horizontal lines indicate corresponding 95% confidence intervals around RRs. Regression coefficients from the sex-specific models were multiplied by the reciprocal of the sex-specific regression dilution ratio to correct for regression dilution bias. The reference category for SBP was 110-115 mmHg in both sexes separately. The reference category for DBP was 70-<75 mmHg in both sexes separately. Abbreviations: CVD, cardiovascular disease; DBP, diastolic blood pressure; mmHg, millimetres of mercury; RR, relative risk; SBP, systolic blood pressure.

# SUPPLEMENTAL TABLES

Table S1. Missing data in the sample for the current study.

| **Variable** | **Missing, n (%)** |
| --- | --- |
| Sex | 0 (0.0%) |
| Age | 0 (0.0%) |
| Townsend deprivation index | 497 (0.1%) |
| Smoking status | 2,062 (0.5%) |
| Diabetes | 1,434 (0.3%) |
| Antihypertensive medication use | 0 (0.0%) |
| Lipid-lowering medication use | 0 (0.0%) |
| BMI | 0 (0.0%) |
| Total cholesterol | 25,481 (6.1%) |
| HDL-C | 59,032 (14.0%) |
| Menopausal status^a^ | 21,391 (9.1%) |
| Abbreviations: BMI, body mass index; HDL-C, high-density lipoprotein cholesterol. | |
| ^a^Calculated as % missing from women participants only. | |

Table S2. Baseline characteristics of 420,649 UK Biobank participants included in the main analyses stratified by AHA hypertension category.

| **Characteristics** | | | **Overall** | |  | **Women** | |  | **Men** | |
| --- | --- | --- | --- | --- | --- | --- | --- | --- | --- | --- |
|  |  |  | **No hypertension** | **Hypertension** |  | **No hypertension** | **Hypertension** |  | **No hypertension** | **Hypertension** |
|  |  |  | **(n = 116,032)** | **(n = 304,617)** |  | **(n = 79,701)** | **(n = 155,855)** |  | **(n = 36,331)** | **(n = 148,762)** |
| Incident CVD cases during follow-up, n | | | 4,283 | 24,345 |  | 1,969 | 8,362 |  | 2,314 | 15,983 |
| Age, mean (SD) | | | 53.3 (8.1) | 57.2 (7.8) |  | 53.2 (8.0) | 57.6 (7.6) |  | 53.5 (8.3) | 56.8 (8.1) |
| Ethnicity | | |  |  |  |  |  |  |  |  |
|  | White | | 108,299 (93.8%) | 286,724 (94.6%) |  | 74,891 (94.4%) | 146,606 (94.4%) |  | 33,408 (92.5%) | 140,118 (94.7%) |
|  | Other^a^ | | 7,165 (6.2%) | 16,422 (5.4%) |  | 4,473 (5.6%) | 8,623 (5.6%) |  | 2,692 (7.5%) | 7,799 (5.3%) |
| Socioeconomic status | | |  |  |  |  |  |  |  |  |
|  | Townsend deprivation index score, median (IQR) | | -2.0 (-3.6, -0.7) | -2.2 (-3.7, -0.3) |  | -2.1 (-3.6, -0.5) | -2.2 (-3.7, -0.3) |  | -2.0 (-3.6, -1.0) | -2.2 (-3.7, -0.4) |
|  | Townsend deprivation fifths: | |  |  |  |  |  |  |  |  |
|  |  | First (least deprived) | 42,003 (36.2%) | 116,073 (38.1%) |  | 29,131 (36.6%) | 59,218 (38.0%) |  | 12,872 (35.4%) | 56,855 (38.2%) |
|  |  | Second | 23,106 (19.9%) | 63,169 (20.7%) |  | 16,076 (20.2%) | 32,744 (21.0%) |  | 7,030 (19.4%) | 30,425 (20.5%) |
|  |  | Third | 17,719 (15.3%) | 45,169 (14.8%) |  | 12,423 (15.6%) | 23,330 (15.0%) |  | 5,296 (14.6%) | 21,839 (14.7%) |
|  |  | Fourth | 16,249 (14.0%) | 39,784 (13.1%) |  | 11,045 (13.9%) | 20,576 (13.2%) |  | 5,204 (14.3%) | 19,208 (12.9%) |
|  |  | Fifth (most deprived) | 16,955 (14.6%) | 40,422 (13.3%) |  | 11,026 (13.8%) | 19,987 (12.8%) |  | 5,929 (16.3%) | 20,435 (13.7%) |
| Smoking status: | | |  |  |  |  |  |  |  |  |
|  | Never | | 66,533 (57.6%) | 167,665 (55.3%) |  | 47,044 (59.3%) | 93,970 (60.6%) |  | 19,489 (53.9%) | 73,695 (49.8%) |
|  | Previous | | 35,196 (30.5%) | 105,903 (34.9%) |  | 23,926 (30.2%) | 49,052 (31.6%) |  | 11,270 (31.2%) | 56,851 (38.4%) |
|  | Current light (<15 cigarettes/day) | | 4,667 (4.0%) | 8,348 (2.8%) |  | 3,229 (4.1%) | 4,469 (2.9%) |  | 1,438 (4.0%) | 3,879 (2.6%) |
|  | Current medium (15-<30 cigarettes/day) | | 4,353 (3.8%) | 9,956 (3.3%) |  | 2,755 (3.5%) | 4,306 (2.8%) |  | 1,598 (4.4%) | 5,650 (3.8%) |
|  | Current heavy (≥30 cigarettes/day) | | 4,732 (4.1%) | 11,234 (3.7%) |  | 2,387 (3.0%) | 3,289 (2.1%) |  | 2,345 (6.5%) | 7,945 (5.4%) |
| Diabetes | | | 11,279 (9.8%) | 36,504 (12.0%) |  | 7,329 (9.2%) | 17,413 (11.2%) |  | 3,950 (10.9%) | 19,091 (12.9%) |
| Medication use: | | |  |  |  |  |  |  |  |  |
|  | Antihypertensive medication use | | 12,565 (10.8%) | 82,734 (27.2%) |  | 7,392 (9.3%) | 39,873 (25.6%) |  | 5,173 (14.2%) | 42,861 (28.8%) |
|  | Lipid-lowering medication use | | 9,144 (7.9%) | 47,393 (15.6%) |  | 4,969 (6.2%) | 19,790 (12.7%) |  | 4,175 (11.5%) | 27,603 (18.6%) |
| Blood Pressure (mmHg): | | |  |  |  |  |  |  |  |  |
|  | SBP, mean (SD) | | 71.9 (5.3) | 86.3 (8.5) |  | 71.6 (5.4) | 85.4 (8.3) |  | 72.7 (5.0) | 87.3 (8.6) |
|  | DBP, mean (SD) | | 87.1 (5.4) | 106.0 (9.4) |  | 86.5 (5.5) | 105.1 (9.2) |  | 88.5 (4.8) | 106.9 (9.4) |
| BMI (kg/m^2^): | | |  |  |  |  |  |  |  |  |
|  | BMI, mean (SD) | | 25.5 (4.1) | 27.9 (4.8) |  | 25.2 (4.2) | 27.8 (5.3) |  | 26.0 (3.7) | 28.1 (4.2) |
|  | Overweight (25-<30) | | 42,773 (36.9%) | 136,170 (44.7%) |  | 26,377 (33.1%) | 60,526 (38.8%) |  | 16,396 (45.1%) | 75,644 (50.8%) |
|  | Obesity (≥30) | | 14,285 (12.3%) | 84,467 (27.7%) |  | 9,643 (12.1%) | 44,193 (28.4%) |  | 4,642 (12.8%) | 40,274 (27.1%) |
| Serum lipids (mmol/L): | | |  |  |  |  |  |  |  |  |
|  | Total cholesterol, mean (SD) | | 5.6 (1.1) | 5.8 (1.1) |  | 5.7 (1.1) | 6.0 (1.1) |  | 5.4 (1.0) | 5.6 (1.1) |
|  | HDL-C, mean (SD) | | 1.5 (0.4) | 1.4 (0.4) |  | 1.6 (0.4) | 1.6 (0.4) |  | 1.3 (0.3) | 1.3 (0.3) |
| Numbers are participant numbers (%), with % representing the column percentage estimated excluding participants with missing responses, unless otherwise specified. | | | | | | | | | | |
| Abbreviations: AHA, American Heart Association; BMI, body mass index; CVD, cardiovascular disease; DBP, diastolic blood pressure; HDL-C, high-density lipoprotein cholesterol; IQR, interquartile range; kg/m^2^, kilogram per square meter; mmHg, millimetres of mercury; mmol/L, millimoles per litre; SBP, systolic blood pressure; SD, standard deviation. | | | | | | | | | | |
| ^a^Includes Asian or Asian British, black or black British, Caribbean, African, any other black background, Chinese, other ethnic group, white and black Caribbean, white and black African, white and Asian, any other mixed background, Indian, Pakistani, Bangladeshi, any other Asian background. | | | | | | | | | | |

Table S3. Baseline characteristics of 233,556 women in UK Biobank in the main analyses stratified by SBP categories.

| **Characteristics** | | | **SBP range (mmHg)** | | | | | | | | | |
| --- | --- | --- | --- | --- | --- | --- | --- | --- | --- | --- | --- | --- |
|  |  |  | **<100** | **100-<110** | **110-<120** | **120-<130** | **130-<140** | **140-<150** | **150-<160** | **160-<170** | **170-<180** | **≥180** |
|  |  |  | **(n=2,309)** | **(n=14,638)** | **(n=35,970)** | **(n=48,864)** | **(n=47,113)** | **(n=36,636)** | **(n=24,468)** | **(n=13,798)** | **(n=6,973)** | **(n=4,787)** |
| Incident CVD cases during follow-up, n | | | 39 | 223 | 784 | 1,556 | 2,063 | 1,980 | 1,558 | 1,030 | 588 | 510 |
| Age, mean (SD) | | | 50.1  (7.4) | 50.6  (7.4) | 52.2  (7.7) | 54.4  (7.8) | 56.6  (7.6) | 58.4  (7.1) | 59.7  (6.7) | 60.5  (6.4) | 61.2  (6.1) | 61.6  (6.1) |
| Ethnicity | | |  |  |  |  |  |  |  |  |  |  |
|  | White | | 2,098 (91.5%) | 13,535 (92.9%) | 33,570 (93.7%) | 45,939 (94.4%) | 44,452 (94.7%) | 34,582 (94.8%) | 23,143 (94.9%) | 13,078 (95.1%) | 6,594 (94.9%) | 4,506 (94.4%) |
|  | Other^a^ | | 194  (8.5%) | 1,043 (7.2%) | 2,258 (6.3%) | 2,712 (5.6%) | 2,472  (5.3%) | 1,885  (5.2%) | 1,239  (5.1%) | 673  (4.9%) | 354  (5.1%) | 266  (5.6%) |
| Socioeconomic status | | |  |  |  |  |  |  |  |  |  |  |
|  | Townsend deprivation index score, median (IQR) | | -1.5  (-3.3, -1.6) | -1.9  (-3.5, -0.9) | -2.0  (-3.6, -0.7) | -2.1  (-3.6, -0.5) | -2.2  (-3.7, -0.3) | -2.2  (-3.7, -0.2) | -2.3  (-3.7, -0.0) | -2.4  (-3.7, -0.1) | -2.4  (-3.7, -0.1) | -2.4  (-3.8, 0.1) |
|  | Townsend deprivation fifths: | |  |  |  |  |  |  |  |  |  |  |
|  |  | First (least deprived) | 698 (30.2%) | 5,058 (34.6%) | 12,874 (35.8%) | 18,032 (36.9%) | 17,827 (37.8%) | 13,983 (38.2%) | 9,635 (39.4%) | 5,499 (39.9%) | 2,787 (40.0%) | 1,956 (40.9%) |
|  |  | Second | 449 (19.5%) | 2,830 (19.3%) | 7,157 (19.9%) | 9,991 (20.5%) | 9,809 (20.8%) | 7,823 (21.4%) | 5,345 (21.8%) | 2,904 (21.1%) | 1,475 (21.2%) | 1,037 (21.7%) |
|  |  | Third | 353 (15.3%) | 2,347 (16.0%) | 5,624 (15.6%) | 7,539 (15.4%) | 7,115 (15.1%) | 5,515 (15.1%) | 3,597 (14.7%) | 1,971 (14.3%) | 1,001 (14.4%) | 691  (14.4%) |
|  |  | Fourth | 360 (15.6%) | 2,161 (14.8%) | 5,124 (14.3%) | 6,640 (13.6%) | 6,311 (13.4%) | 4,725 (12.9%) | 3,036 (12.4%) | 1,767 (12.8%) | 917  (13.2%) | 580  (12.1%) |
|  |  | Fifth (most deprived) | 449 (19.5%) | 2,242 (15.3%) | 5,191 (14.4%) | 6,662 (13.6%) | 6,051 (12.8%) | 4,590 (12.5%) | 2,855 (11.7%) | 1,657 (12.0%) | 793  (11.4%) | 523  (10.9%) |
| Smoking status: | | |  |  |  |  |  |  |  |  |  |  |
|  | Never | | 1,334 (58.2%) | 8,485 (58.2%) | 21,174 (59.1%) | 29,150 (59.9%) | 28,249 (60.3%) | 22,030 (60.4%) | 14,856 (61.0%) | 8,425 (61.4%) | 4,333 (62.5%) | 2,978 (62.6%) |
|  | Previous | | 626 (27.3%) | 4,299 (29.5%) | 10,752 (30.0%) | 14,761 (30.3%) | 14,844 (31.7%) | 11,624 (31.9%) | 7,877 (32.4%) | 4,461 (32.5%) | 2,202 (31.8%) | 1,532 (32.2%) |
|  | Current light (<15 cigarettes/day) | | 131  (5.7%) | 719  (4.9%) | 1,462 (4.1%) | 1,780 (3.7%) | 1,428  (3.1%) | 1,041  (2.9%) | 626  (2.6%) | 288  (2.1%) | 134  (1.9%) | 89  (1.9%) |
|  | Current medium (15-<30 cigarettes/day) | | 97  (4.2%) | 563  (3.9%) | 1,307 (3.7%) | 1,605 (3.3%) | 1,319  (2.8%) | 1,011  (2.8%) | 597  (2.5%) | 316  (2.3%) | 154  (2.2%) | 92  (1.9%) |
|  | Current heavy (≥30 cigarettes/day) | | 103  (4.5%) | 517  (3.6%) | 1,113 (3.1%) | 1,352 (2.8%) | 1,041  (2.2%) | 743  (2.0%) | 393  (1.6%) | 239  (1.7%) | 112  (1.6%) | 63  (1.3%) |
| Diabetes | | | 208  (9.0%) | 1,179 (8.1%) | 3,220 (9.0%) | 4,890 (10.0%) | 5,097 (10.9%) | 4,277 (11.7%) | 2,879 (11.8%) | 1,632 (11.9%) | 804  (11.6%) | 556  (11.7%) |
| Medication use: | | |  |  |  |  |  |  |  |  |  |  |
|  | Antihypertensive medication use | | 97  (4.2%) | 734  (5.0%) | 2,966 (8.3%) | 6,893 (14.1%) | 9,995 (21.2%) | 10,065 (27.5%) | 7,833 (32.0%) | 4,660 (33.8%) | 2,390 (34.3%) | 1,632 (34.1%) |
|  | Lipid-lowering medication use | | 75  (3.3%) | 538  (3.7%) | 1,964 (5.5%) | 4,091 (8.4%) | 5,327 (11.3%) | 5,197 (14.2%) | 3,676 (15.0%) | 2,159 (15.7%) | 1,016 (14.6%) | 716  (15.0%) |
| Blood Pressure (mmHg): | | |  |  |  |  |  |  |  |  |  |  |
|  | SBP, mean (SD) | | 96.3  (3.1) | 105.9  (2.7) | 115.2  (2.8) | 124.8  (2.9) | 134.6  (2.9) | 144.5  (2.9) | 154.4  (2.9) | 164.2  (2.8) | 174.1  (2.8) | 190.1  (10.0) |
|  | DBP, mean (SD) | | 62.2  (4.8) | 67.8  (5.1) | 72.9  (5.7) | 77.6  (6.3) | 81.6  (7.0) | 85.0  (7.7) | 87.8  (8.3) | 90.4  (8.9) | 92.8  (9.3) | 96.9  (10.5) |
|  | Hypertension | | 0  (0.0%) | 120  (0.8%) | 3,926 (10.9%) | 18,034 (36.9%) | 47,113 (100.0%) | 36,636 (100.0%) | 24,468 (100.0%) | 13,798 (100.0%) | 6,973 (100.0%) | 4,787 (100.0%) |
| BMI (kg/m^2^): | | |  |  |  |  |  |  |  |  |  |  |
|  | BMI, mean (SD) | | 23.0  (3.4) | 24.3  (3.8) | 25.6  (4.5) | 26.6  (4.9) | 27.5  (5.1) | 27.9  (5.3) | 28.0  (5.3) | 28.0  (5.2) | 28.1  (5.3) | 28.0  (5.2) |
|  | Overweight (25-<30) | | 431 (18.7%) | 4,047 (27.6%) | 12,037 (33.5%) | 17,927 (36.7%) | 17,951 (38.1%) | 14,235 (38.9%) | 9,777 (40.0%) | 5,699 (41.3%) | 2,784 (39.9%) | 2,015 (42.1%) |
|  | Obesity (≥30) | | 79  (3.4%) | 950  (6.5%) | 3,977 (11.1%) | 7,694 (15.7%) | 9,320 (19.8%) | 7,984 (21.8%) | 5,420 (22.2%) | 2,996 (21.7%) | 1,590 (22.8%) | 1,034 (21.6%) |
| Serum lipids (mmol/L): | | |  |  |  |  |  |  |  |  |  |  |
|  | Total cholesterol, mean (SD) | | 5.4  (1.0) | 5.5  (1.0) | 5.7  (1.1) | 5.8  (1.1) | 5.9  (1.1) | 6.0  (1.1) | 6.1  (1.1) | 6.2  (1.1) | 6.2  (1.1) | 6.3  (1.2) |
|  | HDL-C, mean (SD) | | 1.6  (0.4) | 1.6  (0.4) | 1.6  (0.4) | 1.6  (0.4) | 1.6  (0.4) | 1.6  (0.4) | 1.6  (0.4) | 1.6  (0.4) | 1.6  (0.4) | 1.6  (0.4) |
| Numbers are participant numbers (%), with % representing the column percentage estimated excluding participants with missing responses, unless otherwise specified. | | | | | | | | | | | | |
| Abbreviations: BMI, body mass index; CVD, cardiovascular disease; DBP, diastolic blood pressure; HDL-C, high-density lipoprotein cholesterol; IQR, interquartile range; kg/m^2^, kilogram per square meter; mmHg, millimetres of mercury; mmol/L, millimoles per litre; SBP, systolic blood pressure; SD, standard deviation. | | | | | | | | | | | | |
| ^a^Includes Asian or Asian British, black or black British, Caribbean, African, any other black background, Chinese, other ethnic group, white and black Caribbean, white and black African, white and Asian, any other mixed background, Indian, Pakistani, Bangladeshi, any other Asian background. | | | | | | | | | | | | |

Table S4. Baseline characteristics of 185,093 men in UK Biobank in the main analyses stratified by SBP categories.

| **Characteristics** | | | **SBP range (mmHg)** | | | | | | | | | |
| --- | --- | --- | --- | --- | --- | --- | --- | --- | --- | --- | --- | --- |
|  |  |  | **<100** | **100-<110** | **110-<120** | **120-<130** | **130-<140** | **140-<150** | **150-<160** | **160-<170** | **170-<180** | **≥180** |
|  |  |  | **(n=275)** | **(n=2,641)** | **(n=13,957)** | **(n=33,392)** | **(n=43,997)** | **(n=39,042)** | **(n=26,170)** | **(n=14,315)** | **(n=6,838)** | **(n=4,466)** |
| Incident CVD cases during follow-up, n | | | 28 | 158 | 828 | 2,284 | 3,686 | 4,169 | 3,189 | 2,036 | 1,123 | 796 |
| Age, mean (SD) | | | 54.5  (7.9) | 52.9  (8.1) | 52.7  (8.1) | 53.5  (8.2) | 55.0  (8.2) | 56.8  (7.9) | 58.5  (7.5) | 59.7  (7.0) | 60.5  (6.6) | 61.2  (6.3) |
| Ethnicity | | |  |  |  |  |  |  |  |  |  |  |
|  | White | | 241  (88.9%) | 2,330  (89.3%) | 12,667  (91.3%) | 30,837  (93.0%) | 41,238  (94.3%) | 36,878  (95.0%) | 24,860  (95.5%) | 13,707  (96.2%) | 6,520  (95.9%) | 4,248  (95.6%) |
|  | Other^a^ | | 30  (11.1%) | 279  (10.7%) | 1,202  (8.7%) | 2,340  (7.1%) | 2,514  (5.8%) | 1,936  (5.0%) | 1,170  (4.5%) | 547  (3.8%) | 277  (4.1%) | 196  (4.4%) |
| Socioeconomic status | | |  |  |  |  |  |  |  |  |  |  |
|  | Townsend deprivation index score, median (IQR) | | -0.7  (-3.2, -2.4) | -1.3  (-3.3, -1.9) | -1.8  (-3.5, -1.2) | -2.0  (-3.6, -0.8) | -2.2  (-3.7, -0.5) | -2.2  (-3.7, -0.3) | -2.3  (-3.7, -0.2) | -2.3  (-3.7, -0.2) | -2.3  (-3.8, -0.2) | -2.2  (-3.7, -0.4) |
|  | Townsend deprivation fifths: | |  |  |  |  |  |  |  |  |  |  |
|  |  | First (least deprived) | 76  (27.6%) | 788  (29.8%) | 4,712  (33.8%) | 12,108  (36.3%) | 16,737  (38.0%) | 14,904  (38.2%) | 10,378  (39.7%) | 5,590  (39.1%) | 2,744  (40.1%) | 1,690  (37.8%) |
|  |  | Second | 45  (16.4%) | 487  (18.4%) | 2,638  (18.9%) | 6,581  (19.7%) | 8,802  (20.0%) | 8,099  (20.7%) | 5,454  (20.8%) | 3,018  (21.1%) | 1,402  (20.5%) | 929  (20.8%) |
|  |  | Third | 32  (11.6%) | 364  (13.8%) | 2,123  (15.2%) | 4,856  (14.5%) | 6,567  (14.9%) | 5,761  (14.8%) | 3,730  (14.3%) | 2,087  (14.6%) | 960  (14.0%) | 655  (14.7%) |
|  |  | Fourth | 55  (20.0%) | 413  (15.6%) | 2,004  (14.4%) | 4,737  (14.2%) | 5,689  (12.9%) | 5,035  (12.9%) | 3,292  (12.6%) | 1,794  (12.5%) | 834  (12.2%) | 559  (12.5%) |
|  |  | Fifth (most deprived) | 67  (24.4%) | 589  (22.3%) | 2,480  (17.8%) | 5,110  (15.3%) | 6,202  (14.1%) | 5,243  (13.4%) | 3,316  (12.7%) | 1,826  (12.8%) | 898  (13.1%) | 633  (14.2%) |
| Smoking status: | | |  |  |  |  |  |  |  |  |  |  |
|  | Never | | 135  (49.3%) | 1,388  (52.9%) | 7,515  (54.2%) | 17,886  (53.8%) | 22,790  (52.0%) | 19,522  (50.2%) | 12,354  (47.5%) | 6,567  (46.1%) | 3,084  (45.4%) | 1,943  (43.7%) |
|  | Previous | | 77  (28.1%) | 769  (29.3%) | 4,120  (29.7%) | 10,744  (32.3%) | 15,474  (35.3%) | 14,920  (38.4%) | 10,889 (41.9%) | 6,146  (43.1%) | 2,981  (43.9%) | 2,001  (45.0%) |
|  | Current light (<15 cigarettes/day) | | 13  (4.7%) | 125  (4.8%) | 611  (4.4%) | 1,126  (3.4%) | 1,229  (2.8%) | 994  (2.6%) | 607  (2.3%) | 355  (2.5%) | 163  (2.4%) | 94  (2.1%) |
|  | Current medium (15-<30 cigarettes/day) | | 21  (7.7%) | 157  (6.0%) | 674  (4.9%) | 1,413  (4.3%) | 1,761  (4.0%) | 1,422  (3.7%) | 886  (3.4%) | 509  (3.6%) | 225  (3.3%) | 180  (4.1%) |
|  | Current heavy (≥30 cigarettes/day) | | 28  (10.2%) | 184  (7.0%) | 958  (6.9%) | 2,050  (6.2%) | 2,544  (5.8%) | 2,009  (5.2%) | 1,274  (4.9%) | 676  (4.7%) | 340  (5.0%) | 227  (5.1%) |
| Diabetes | | | 208  (9.0%) | 47  (17.3%) | 299  (11.4%) | 1,483  (10.7%) | 3,680  (11.1%) | 5,434  (12.4%) | 5,161  (13.3%) | 3,525  (13.5%) | 1,950  (13.7%) | 871  (12.8%) |
| Medication use: | | |  |  |  |  |  |  |  |  |  |  |
|  | Antihypertensive medication use | | 63  (22.9%) | 325  (12.3%) | 1,786  (12.8%) | 5,639  (16.9%) | 10,110  (23.0%) | 11,631  (29.8%) | 9,200  (35.2%) | 5,255  (36.7%) | 2,496  (36.5%) | 1,529  (34.2%) |
|  | Lipid-lowering medication use | | 53  (19.3%) | 266  (10.1%) | 1,418  (10.2%) | 4,233  (12.7%) | 6,984  (15.9%) | 7,483  (19.2%) | 5,811  (22.2%) | 3,183  (22.2%) | 1,469  (21.5%) | 878  (19.7%) |
| Blood Pressure (mmHg): | | |  |  |  |  |  |  |  |  |  |  |
|  | SBP, mean (SD) | | 96.2  (3.7) | 106.5  (2.5) | 115.8  (2.8) | 125.2  (2.8) | 134.8  (2.9) | 144.5  (2.9) | 154.3  (2.9) | 164.2  (2.8) | 174.0  (2.8) | 189.7  (9.7) |
|  | DBP, mean (SD) | | 61.2  (6.1) | 67.4  (5.3) | 72.7  (5.7) | 77.8  (6.1) | 82.6  (6.5) | 86.7  (7.1) | 90.2  (7.8) | 93.2  (8.5) | 96.0  (9.3) | 100.7  (10.8) |
|  | Hypertension | | 0  (0.0%) | 25  (1.0%) | 1,438  (10.3%) | 12,471  (37.4%) | 43,997  (100.0%) | 39,042  (100.0%) | 26,170  (100.0%) | 14,315  (100.0%) | 6,838  (100.0%) | 4,466  (100.0%) |
| BMI (kg/m^2^): | | |  |  |  |  |  |  |  |  |  |  |
|  | BMI, mean (SD) | | 24.4  (4.4) | 24.9  (3.8) | 26.0  (3.9) | 26.9  (4.0) | 27.7  (4.1) | 28.2  (4.2) | 28.4  (4.2) | 28.4  (4.1) | 28.5  (4.3) | 28.5  (4.2) |
|  | Overweight (25-<30) | | 83  (30.2%) | 901  (34.1%) | 6,070  (43.5%) | 16,250  (48.7%) | 22,271  (50.6%) | 19,984  (51.2%) | 13,427  (51.3%) | 7,382  (51.6%) | 3,481  (50.9%) | 2,191  (49.1%) |
|  | Obesity (≥30) | | 29  (10.5%) | 197  (7.5%) | 1,493  (10.7%) | 4,810  (14.4%) | 7,967  (18.1%) | 8,120  (20.8%) | 5,733  (21.9%) | 3,212  (22.4%) | 1,549  (22.7%) | 1,075  (24.1%) |
| Serum lipids (mmol/L): | | |  |  |  |  |  |  |  |  |  |  |
|  | Total cholesterol, mean (SD) | | 5.1  (1.1) | 5.3  (1.0) | 5.4  (1.0) | 5.5  (1.1) | 5.6  (1.1) | 5.6  (1.1) | 5.6  (1.1) | 5.7  (1.1) | 5.7  (1.1) | 5.8  (1.1) |
|  | HDL-C, mean (SD) | | 1.3  (0.3) | 1.3  (0.3) | 1.3  (0.3) | 1.3  (0.3) | 1.3  (0.3) | 1.3  (0.3) | 1.3  (0.3) | 1.3  (0.3) | 1.3  (0.3) | 1.4  (0.4) |
| Numbers are participant numbers (%), with % representing the column percentage estimated excluding participants with missing responses, unless otherwise specified. | | | | | | | | | | | | |
| Abbreviations: BMI, body mass index; CVD, cardiovascular disease; DBP, diastolic blood pressure; HDL-C, high-density lipoprotein cholesterol; IQR, interquartile range; kg/m^2^ , kilogram per square meter; mmHg, millimetres of mercury; mmol/L, millimoles per litre; SBP, systolic blood pressure; SD, standard deviation. | | | | | | | | | | | | |
| ^a^Includes Asian or Asian British, black or black British, Caribbean, African, any other black background, Chinese, other ethnic group, white and black Caribbean, white and black African, white and Asian, any other mixed background, Indian, Pakistani, Bangladeshi, any other Asian background. | | | | | | | | | | | | |

Table S5. Risks and sex-specific RDs for BP and CVD by sex.

| **Blood pressure category** | |  | **Risks per 10,000 person-years (95% CI)** | |  | **RD (95% CI)** | |
| --- | --- | --- | --- | --- | --- | --- | --- |
|  |  |  | **Women** | **Men** |  | **Women** | **Men** |
| **SBP, mmHg** | |  |  |  |  |  |  |
|  | <100 |  | 19.20 (13.17, 25.24) | 85.02 (53.53, 116.52) |  | -1.56 (-7.60, 4.48) | 37.83 (6.33, 69.32) |
|  | 100-<105 |  | 15.60 (11.82, 19.37) | 61.95 (43.21, 80.69) |  | -5.17 (-8.94, -1.39) | 14.75 (-3.98, 33.49) |
|  | 105-<110 |  | 17.30 (14.58, 20.02) | 51.08 (41.78, 60.39) |  | -3.47 (-6.19, -0.74) | 3.89 (-5.41, 13.19) |
|  | 110-<115 (Ref) | | 20.76 (18.46, 23.07) | 47.19 (41.36, 53.03) |  | Ref | Ref |
|  | 115-<120 |  | 21.59 (19.62, 23.56) | 56.65 (52.01, 61.29) |  | 0.83 (-1.14, 2.80) | 9.46 (4.81, 14.10) |
|  | 120-<125 |  | 25.81 (23.89, 27.73) | 58.03 (54.33, 61.73) |  | 5.05 (3.13, 6.97) | 10.84 (7.14, 14.54) |
|  | 125-<130 |  | 27.85 (25.98, 29.72) | 60.36 (57.11, 63.62) |  | 7.08 (5.21, 8.96) | 13.17 (9.91, 16.42) |
|  | 130-<135 |  | 31.55 (29.61, 33.49) | 65.65 (62.55, 68.75) |  | 10.78 (8.84, 12.72) | 18.45 (15.35, 21.56) |
|  | 135-<140 |  | 33.13 (31.13, 35.14) | 69.98 (66.88, 73.09) |  | 12.37 (10.36, 14.38) | 22.79 (19.69, 25.89) |
|  | 140-<145 |  | 34.73 (32.59, 36.87) | 77.24 (73.95, 80.52) |  | 13.97 (11.83, 16.11) | 30.04 (26.76, 33.32) |
|  | 145-<155 |  | 37.75 (35.36, 40.14) | 83.39 (79.77, 87.01) |  | 16.99 (14.60, 19.38) | 36.19 (32.57, 39.82) |
|  | 150-<155 |  | 40.01 (37.35, 42.68) | 84.04 (80.09, 87.99) |  | 19.25 (16.58, 21.92) | 36.85 (32.90, 40.79) |
|  | 155-<160 |  | 38.74 (35.83, 41.66) | 87.91 (83.35, 92.46) |  | 17.98 (15.07, 20.89) | 40.71 (36.16, 45.27) |
|  | 160-<165 |  | 43.13 (39.60, 46.67) | 97.63 (92.09, 103.17) |  | 22.37 (18.84, 25.90) | 50.44 (44.89, 55.98) |
|  | 165-<170 |  | 46.62 (42.30, 50.93) | 93.42 (87.06, 99.78) |  | 25.85 (21.54, 30.17) | 46.22 (39.86, 52.58) |
|  | 170-<175 |  | 50.09 (44.88, 55.31) | 112.54 (104.18, 120.90) |  | 29.33 (24.11, 34.55) | 65.35 (56.99, 73.71) |
|  | 175-<180 |  | 46.49 (40.48, 52.50) | 102.00 (92.24, 111.76) |  | 25.73 (19.71, 31.74) | 54.81 (45.05, 64.56) |
|  | ≥180 |  | 60.53 (55.24, 65.82) | 114.98 (106.94, 123.01) |  | 39.77 (34.47, 45.06) | 67.78 (59.75, 75.82) |
| **DBP, mmHg** | |  |  |  |  |  |  |
|  | <60 |  | 31.36 (24.88, 37.83) | 77.84 (59.06, 96.61) |  | 4.12 (-2.36, 10.60) | 13.50 (-5.28, 32.28) |
|  | 60-<65 |  | 22.74 (19.71, 25.77) | 66.55 (57.49, 75.62) |  | -4.49 (-7.53, -1.46) | 2.22 (-6.85, 11.28) |
|  | 65-<70 |  | 26.68 (24.65, 28.71) | 63.25 (58.37, 68.12) |  | -0.55 (-2.58, 1.47) | -1.09 (-5.97, 3.78) |
|  | 70-<75 (Ref) |  | 27.23 (25.72, 28.75) | 64.34 (61.10, 67.57) |  | Ref | Ref |
|  | 75-<80 |  | 31.37 (29.95, 32.78) | 67.93 (65.35, 70.51) |  | 4.13 (2.72, 5.55) | 3.59 (1.01, 6.17) |
|  | 80-<85 |  | 33.01 (31.57, 34.44) | 70.10 (67.75, 72.46) |  | 5.77 (4.34, 7.21) | 5.77 (3.41, 8.12) |
|  | 85-<90 |  | 35.29 (33.62, 36.97) | 76.01 (73.49, 78.53) |  | 8.06 (6.38, 9.73) | 11.67 (9.15, 14.20) |
|  | 90-<95 |  | 39.38 (37.19, 41.58) | 86.26 (83.12, 89.39) |  | 12.15 (9.95, 14.35) | 21.92 (18.78, 25.06) |
|  | 95-<100 |  | 42.23 (39.01, 45.45) | 93.15 (88.85, 97.46) |  | 14.99 (11.78, 18.21) | 28.81 (24.51, 33.12) |
|  | ≥100 |  | 55.01 (50.54, 59.48) | 108.24 (102.93, 113.54) |  | 27.78 (23.31, 32.25) | 43.90 (38.60, 49.20) |
| **Hypertension** | |  |  |  |  |  |  |
|  | No hypertension | | 22.86 (21.83, 23.88) | 55.61 (53.33, 57.90) |  | Ref | Ref |
|  | Hypertension |  | 37.12 (36.32, 37.92) | 80.88 (79.62, 82.13) |  | 14.26 (13.46, 15.06) | 25.26 (24.01, 26.52) |
| Abbreviations: CI, confidence intervals; CVD, cardiovascular disease; DBP, diastolic blood pressure; mmHg, millimetres of mercury; RD, risk difference; Ref, reference category; SBP, systolic blood pressure. | | | | | | | |

Table S6. Risks and sex-specific RDs for BP and CHD by sex.

| **Blood pressure category** | |  | **Risks per 10,000 person-years (95% CI)** | |  | **RD (95% CI)** | |
| --- | --- | --- | --- | --- | --- | --- | --- |
|  |  |  | **Women** | **Men** |  | **Women** | **Men** |
| **SBP, mmHg** | |  |  |  |  |  |  |
|  | <100 |  | 12.18 (7.49, 16.87) | 71.52 (42.90, 100.13) |  | -0.24 (-4.93, 4.45) | 34.89 (6.28, 63.51) |
|  | 100-<105 |  | 5.84 (3.59, 8.09) | 53.57 (36.30, 70.83) |  | -6.57 (-8.82, -4.32) | 16.94 (-0.32, 34.21) |
|  | 105-<110 |  | 10.54 (8.47, 12.62) | 34.33 (26.80, 41.85) |  | -1.87 (-3.95, 0.20) | -2.29 (-9.82, 5.23) |
|  | 110-<115 (Ref) | | 12.42 (10.67, 14.17) | 36.62 (31.54, 41.70) |  | Ref | Ref |
|  | 115-<120 |  | 13.97 (12.41, 15.53) | 43.39 (39.38, 47.40) |  | 1.55 (-0.01, 3.11) | 6.77 (2.76, 10.78) |
|  | 120-<125 |  | 16.79 (15.36, 18.32) | 45.06 (41.84, 48.29) |  | 4.37 (2.84, 5.90) | 8.44 (5.21, 11.66) |
|  | 125-<130 |  | 18.06 (16.56, 19.55) | 45.74 (42.93, 48.54) |  | 5.64 (4.15, 7.13) | 9.12 (6.31, 11.92) |
|  | 130-<135 |  | 20.25 (18.70, 21.80 | 52.27 (49.52, 55.02) |  | 7.83 (6.29, 9.38) | 15.65 (12.90, 18.40) |
|  | 135-<140 |  | 22.36 (20.71, 24.01) | 55.20 (52.46, 57.93) |  | 9.94 (8.30, 11.59) | 18.57 (15.84, 21.31) |
|  | 140-<145 |  | 22.84 (21.11, 24.58) | 61.71 (58.79, 64.63) |  | 10.43 (8.69, 12.16) | 25.09 (22.17, 28.01) |
|  | 145-<155 |  | 25.50 (25.53, 27.47) | 63.92 (60.76, 67.07) |  | 13.08 (11.12, 15.05) | 27.30 (24.14, 30.45) |
|  | 150-<155 |  | 26.62 (24.43, 28.80) | 66.22 (62.73, 69.72) |  | 14.20 (12.02, 16.38) | 29.60 (26.11, 33.10) |
|  | 155-<160 |  | 24.11 (21.81, 26.42) | 68.07 (64.06, 72.07) |  | 11.70 (6.39, 14.00) | 31.45 (27.44, 35.45) |
|  | 160-<165 |  | 29.20 (26.28, 32.12) | 75.97 (71.08, 80.85) |  | 16.79 (13.87, 19.71) | 39.35 (34.46, 44.23) |
|  | 165-<170 |  | 31.11 (27.57, 34.65) | 70.65 (65.13, 76.18) |  | 18.69 (15.15, 22.24) | 34.03 (28.51, 39.56) |
|  | 170-<175 |  | 32.86 (28.61, 37.10) | 88.81 (81.39, 96.22) |  | 20.44 (16.19, 24.69) | 52.18 (44.77, 59.60) |
|  | 175-<180 |  | 30.09 (25.23, 34.95) | 76.41 (67.97, 84.84) |  | 17.67 (12.81, 22.53) | 39.79 (31.35, 48.22) |
|  | ≥180 |  | 38.41 (35.12, 43.70) | 85.54 (78.63, 92.46) |  | 26.99 (22.70, 31.29) | 48.92 (42.01, 55.84) |
| **DBP, mmHg** | |  |  |  |  |  |  |
|  | <60 |  | 21.68 (16.32, 27.03) | 66.59 (49.31, 83.88) |  | 3.93 (-1.42, 9.29) | 15.52 (-1.77, 32.80) |
|  | 60-<65 |  | 13.93 (11.57, 16.29) | 50.91 (43.02, 58.80) |  | -3.81 (-6.17, -1.45) | -0.17 (-8.06, 7.72) |
|  | 65-<70 |  | 17.53 (15.90, 19.17) | 47.34 (43.15, 51.52) |  | -0.21 (-1.84, 1.42) | -3.74 (-7.93, 0.45) |
|  | 70-<75 (Ref) |  | 17.74 (16.53, 18.96) | 51.08 (48.21, 53.95) |  | Ref | Ref |
|  | 75-<80 |  | 20.56 (19.42, 21.70) | 53.68 (51.40, 55.96) |  | 2.82 (1.68, 3.96) | 2.60 (0.32, 4.89) |
|  | 80-<85 |  | 21.58 (20.42, 22.74) | 54.21 (52.15, 56.28) |  | 3.84 (2.68, 4.99) | 3.13 (1.07, 5.20) |
|  | 85-<90 |  | 22.97 (21.63, 24.32) | 58.94 (56.73, 61.15) |  | 5.23 (3.88, 6.57) | 7.86 (5.65, 10.07) |
|  | 90-<95 |  | 25.47 (23.71, 27.23) | 67.04 (64.29, 69.78) |  | 7.73 (5.97, 9.49) | 15.96 (13.21, 18.71) |
|  | 95-<100 |  | 27.60 (25.01, 30.19) | 72.68 (68.91, 76.46) |  | 9.85 (7.27, 12.44) | 21.60 (17.83, 25.38) |
|  | ≥100 |  | 33.32 (29.88, 36.77) | 80.84 (76.31, 85.38) |  | 15.58 (12.13, 19.03) | 29.76 (25.23, 34.30) |
| **Hypertension** | |  |  |  |  |  |  |
|  | No hypertension | | 14.52 (13.72, 15.33) | 42.52 (40.54, 44.49) |  | Ref | Ref |
|  | Hypertension |  | 24.29 (23.64, 24.94) | 62.98 (61.88, 64.08) |  | 9.77 (9.12, 10.41) | 20.46 (19.36, 21.56) |
| Abbreviations: CHD, coronary heart disease; CI, confidence intervals; DBP, diastolic blood pressure; mmHg, millimetres of mercury; RD, risk difference; Ref, reference category; SBP, systolic blood pressure. | | | | | | | |

Table S7. Risks and sex-specific RDs for BP and stroke by sex.

| **Blood pressure category** | |  | **Risks per 10,000 person-years (95% CI)** | |  | **RD (95% CI)** | |
| --- | --- | --- | --- | --- | --- | --- | --- |
|  |  |  | **Women** | **Men** |  | **Women** | **Men** |
| **SBP, mmHg** | |  |  |  |  |  |  |
|  | <100 |  | 7.98 (3.94, 12.03) | 15.10 (1.86, 28.34) |  | -0.71 (-4.75, 3.34) | 3.89 (-9.35, 17.13) |
|  | 100-<105 |  | 10.29 (7.09, 13.50) | 8.92 (1.78, 16.06) |  | 1.60 (-1.60, 4.81) | -2.29 (-9.43, 4.85) |
|  | 105-<110 |  | 7.22 (5.40, 9.04) | 17.76 (12.18, 23.33) |  | -1.47 (-3.29, 0.35) | 6.55 (0.97, 12.12) |
|  | 110-<115 (Ref) | | 8.69 (7.15, 10.22) | 11.21 (8.32, 14.10) |  | Ref | Ref |
|  | 115-<120 |  | 7.78 (6.58, 8.99) | 14.06 (11.71, 16.40) |  | -0.91 (-2.11, 0.30) | 2.85 (0.50, 5.19) |
|  | 120-<125 |  | 9.39 (8.22, 10.56) | 13.84 (12.01, 15.66) |  | 0.70 (-0.46, 1.87) | 2.63 (0.80, 4.45) |
|  | 125-<130 |  | 10.19 (9.05, 11.32) | 15.21 (13.57, 16.85) |  | 1.50 (0.36, 2.63) | 4.00 (2.36, 5.64) |
|  | 130-<135 |  | 11.83 (10.65, 13.02) | 14.39 (12.95, 15.84) |  | 3.14 (1.96, 4.33) | 3.18 (1.74, 4.63) |
|  | 135-<140 |  | 11.19 (10.03, 12.34) | 15.05 (13.63, 16.47) |  | 2.50 (1.34, 3.65) | 3.84 (2.42, 5.26) |
|  | 140-<145 |  | 12.30 (11.04, 13.55) | 16.29 (14.82, 17.76) |  | 3.61 (2.35, 4.86) | 5.08 (3.61, 6.55) |
|  | 145-<155 |  | 13.11 (11.74, 14.49) | 20.00 (18.28, 21.72) |  | 4.42 (3.04, 5.80) | 8.79 (7.07, 10.51) |
|  | 150-<155 |  | 13.50 (11.99, 15.01) | 18.65 (16.86, 20.44) |  | 4.81 (3.30, 6.32) | 7.44 (5.65, 9.23) |
|  | 155-<160 |  | 14.89 (13.14, 16.65) | 21.16 (19.03, 23.30) |  | 6.20 (4.45, 7.96) | 9.95 (7.82, 12.09) |
|  | 160-<165 |  | 14.40 (12.42, 16.38) | 23.44 (20.86, 26.02) |  | 5.72 (3.74, 7.69) | 12.23 (9.65, 14.81) |
|  | 165-<170 |  | 15.45 (13.05, 17.86) | 23.01 (20.01, 26.00) |  | 6.76 (4.36, 9.17) | 11.80 (8.80, 14.72) |
|  | 170-<175 |  | 18.17 (15.14, 21.20) | 25.55 (21.81, 29.29) |  | 9.48 (6.45, 12.51) | 14.34 (10.60, 18.09) |
|  | 175-<180 |  | 17.65 (14.07, 21.23) | 26.18 (21.54, 30.82) |  | 8.96 (5.38, 12.54) | 14.97 (10.33, 19.61) |
|  | ≥180 |  | 22.12 (19.05, 25.18) | 30.56 (26.69, 34.42) |  | 13.43 (10.37, 16.49) | 19.35 (15.48, 23.21) |
| **DBP, mmHg** | |  |  |  |  |  |  |
|  | <60 |  | 10.70 (6.93, 14.46) | 14.24 (6.50, 21.98) |  | 0.65 (-3.11, 4.42) | -0.47 (-8.21, 7.27) |
|  | 60-<65 |  | 9.15 (7.23, 11.07) | 15.25 (11.02, 19.47) |  | -0.90 (-2.82, 1.03) | 0.53 (-3.69, 4.76) |
|  | 65-<70 |  | 9.52 (8.31, 10.73) | 16.47 (14.03, 18.91) |  | -0.53 (-1.73, 0.68) | 1.76 (-0.68, 4.20) |
|  | 70-<75 (Ref) |  | 10.05 (9.13, 10.96) | 14.71 (13.20, 16.23) |  | Ref | Ref |
|  | 75-<80 |  | 11.36 (10.41, 12.10) | 15.17 (13.98, 16.36) |  | 1.21 (0.37, 2.05) | 0.46 (-0.74, 1.65) |
|  | 80-<85 |  | 11.68 (10.83, 12.52) | 16.32 (15.21, 17.43) |  | 1.63 (0.73, 2.48) | 1.60 (0.49, 2.72) |
|  | 85-<90 |  | 12.81 (11.81, 13.81) | 18.00 (16.80, 19.20) |  | 2.76 (1.77, 3.76) | 3.28 (2.08, 4.48) |
|  | 90-<95 |  | 14.37 (13.06, 15.69) | 20.37 (18.88, 21.85) |  | 4.33 (3.01, 5.64) | 5.65 (4.17, 7.14) |
|  | 95-<100 |  | 15.13 (13.23, 17.04) | 20.96 (18.97, 22.95) |  | 5.08 (3.18, 6.99) | 6.25 (4.26, 8.24) |
|  | ≥100 |  | 22.18 (19.36, 24.99) | 28.46 (25.81, 31.11) |  | 12.13 (9.32, 14.94) | 13.74 (11.09, 16.04) |
| **Hypertension** | |  |  |  |  |  |  |
|  | No hypertension | | 8.68 (8.05, 9.32) | 13.82 (12.68, 14.96) |  | Ref | Ref |
|  | Hypertension |  | 13.30 (12.83, 13.78) | 18.81 (18.22, 19.40) |  | 4.62 (4.15, 5.09) | 4.99 (4.40, 5.58) |
| Abbreviations: CI, confidence intervals; DBP, diastolic blood pressure; mmHg, millimetres of mercury; RD, risk difference; Ref, reference category; SBP, systolic blood pressure. | | | | | | | |

Table S8. Sex-specific RRs for BP and CVD.

| **Blood pressure category** | |  | **Women** | | |  | **Men** | | |
| --- | --- | --- | --- | --- | --- | --- | --- | --- | --- |
|  |  |  | **Cases** | **Age-adjusted RR (95%CI)** | **Multivariable-adjusted RR (95% CI)^a^** |  | **Cases** | **Age-adjusted RR (95%CI)** | **Multivariable-adjusted RR (95% CI)^a^** |
| **SBP, mmHg** | |  |  |  |  |  |  |  |  |
|  | <100 |  | 39 | 0.93 (0.67, 1.30) | 0.97 (0.67, 1.39) |  | 28 | 1.79 (1.21, 2.65) | 1.81 (1.16, 2.81) |
|  | 100-<105 |  | 66 | 0.76 (0.58, 0.99) | 0.76 (0.57, 1.02) |  | 42 | 1.31 (0.95, 1.82) | 1.47 (1.04, 2.09) |
|  | 105-<110 |  | 157 | 0.84 (0.69, 1.01) | 0.85 (0.69, 1.04) |  | 116 | 1.08 (0.87, 1.35) | 1.23 (0.97, 1.56) |
|  | 110-<115 (Ref) |  | 316 | 1.00 (Ref) | 1.00 (Ref) |  | 252 | 1.00 (Ref) | 1.00 (Ref) |
|  | 115-<120 |  | 468 | 1.04 (0.90, 1.20) | 1.01 (0.86, 1.17) |  | 576 | 1.20 (1.04, 1.39) | 1.25 (1.06, 1.48) |
|  | 120-<125 |  | 702 | 1.24 (1.08, 1.41) | 1.14 (0.99, 1.32) |  | 953 | 1.23 (1.07, 1.41) | 1.27 (1.08, 1.48) |
|  | 125-<130 |  | 854 | 1.33 (1.17, 1.52) | 1.23 (1.07, 1.41) |  | 1,331 | 1.28 (1.12, 1.46) | 1.31 (1.13, 1.53) |
|  | 130-<135 |  | 1,016 | 1.51 (1.33, 1.71) | 1.37 (1.20, 1.58) |  | 1,728 | 1.39 (1.22, 1.59) | 1.40 (1.21, 1.62) |
|  | 135-<140 |  | 1,047 | 1.58 (1.40, 1.80) | 1.38 (1.20, 1.58) |  | 1,958 | 1.48 (1.30, 1.69) | 1.44 (1.25, 1.67) |
|  | 140-<145 |  | 1,015 | 1.66 (1.46, 1.88) | 1.47 (1.28, 1.69) |  | 2,129 | 1.63 (1.43, 1.86) | 1.60 (1.38, 1.85) |
|  | 145-<155 |  | 965 | 1.81 (1.59, 2.05) | 1.57 (1.37, 1.81) |  | 2,040 | 1.76 (1.55, 2.01) | 1.70 (1.47, 1.97) |
|  | 150-<155 |  | 872 | 1.92 (1.68, 2.18) | 1.73 (1.50, 1.99) |  | 1,748 | 1.78 (1.56, 2.03) | 1.70 (1.46, 1.97) |
|  | 155-<160 |  | 686 | 1.86 (1.62, 2.12) | 1.63 (1.41, 1.88) |  | 1,441 | 1.86 (1.63, 2.13) | 1.77 (1.52, 2.06) |
|  | 160-<165 |  | 578 | 2.07 (1.80, 2.38) | 1.84 (1.58, 2.14) |  | 1,202 | 2.07 (1.81, 2.37) | 1.94 (1.66, 2.26) |
|  | 165-<170 |  | 452 | 2.24 (1.94, 2.59) | 2.03 (1.74, 2.38) |  | 834 | 1.98 (1.72, 2.28) | 1.85 (1.58, 2.16) |
|  | 170-<175 |  | 357 | 2.41 (2.07, 2.81) | 2.13 (1.80, 2.52) |  | 701 | 2.39 (2.07, 2.76) | 2.28 (1.94, 2.68) |
|  | 175-<180 |  | 231 | 2.23 (1.88, 2.65) | 1.93 (1.60, 2.32) |  | 422 | 2.17 (1.85, 2.53) | 2.04 (1.72, 2.43) |
|  | ≥180 |  | 510 | 2.92 (2.53, 3.37) | 2.53 (2.16, 2.96) |  | 796 | 2.45 (2.12, 2.82) | 2.31 (1.97, 2.71) |
|  | Per 10 mmHg |  | 10,331 | 1.14 (1.13, 1.15) | 1.12 (1.11, 1.14) |  | 18,297 | 1.12 (1.11, 1.13) | 1.10 (1.09, 1.11) |
| **DBP, mmHg** | |  |  |  |  |  |  |  |  |
|  | <60 |  | 90 | 1.16 (0.93, 1.43) | 1.19 (0.94, 1.52) |  | 66 | 1.22 (0.95, 1.56) | 1.33 (1.01, 1.74) |
|  | 60-<65 |  | 216 | 0.84 (0.72, 0.97) | 0.87 (0.72, 1.02) |  | 207 | 1.04 (0.90, 1.20) | 1.13 (0.97, 1.32) |
|  | 65-<70 |  | 667 | 0.98 (0.89, 1.08) | 1.05 (0.95, 1.16) |  | 647 | 0.98 (0.90, 1.08) | 1.02 (0.93, 1.13) |
|  | 70-<75 (Ref) |  | 1,249 | 1.00 (Ref) | 1.00 (Ref) |  | 1,520 | 1.00 (Ref) | 1.00 (Ref) |
|  | 75-<80 |  | 1,891 | 1.15 (1.07, 1.24) | 1.14 (1.05, 1.23) |  | 2,666 | 1.05 (0.99, 1.12) | 1.04 (0.97, 1.12) |
|  | 80-<85 |  | 2,032 | 1.21 (1.13, 1.30) | 1.15 (1.07, 1.25) |  | 3,399 | 1.09 (1.02, 1.15) | 1.04 (0.97, 1.11) |
|  | 85-<90 |  | 1,708 | 1.29 (1.20, 1.39) | 1.18 (1.08, 1.27) |  | 3,486 | 1.17 (1.11, 1.25) | 1.10 (1.03, 1.17) |
|  | 90-<95 |  | 1,234 | 1.44 (1.33, 1.56) | 1.32 (1.21, 1.44) |  | 2,904 | 1.33 (1.25, 1.42) | 1.22 (1.14, 1.31) |
|  | 95-<100 |  | 662 | 1.54 (1.41, 1.70) | 1.40 (1.26, 1.55) |  | 1,800 | 1.44 (1.34, 1.54) | 1.29 (1.20, 1.39) |
|  | ≥100 |  | 582 | 2.01 (1.82, 2.22) | 1.77 (1.59, 1.98) |  | 1,602 | 1.67 (1.55, 1.79) | 1.47 (1.36, 1.59) |
|  | Per 5 mmHg |  | 10,331 | 1.09 (1.08, 1.10) | 1.07 (1.06, 1.08) |  | 18,297 | 1.08 (1.07, 1.08) | 1.05 (1.04, 1.06) |
| **Hypertension** | |  |  |  |  |  |  |  |  |
|  | No hypertension |  | 1,969 | 1.00 (Ref) | 1.00 (Ref) |  | 2,314 | 1.00 (Ref) | 1.00 (Ref) |
|  | Hypertension |  | 8,362 | 1.62 (1.54, 1.70) | 1.46 (1.38, 1.54) |  | 15,983 | 1.45 (1.39, 1.52) | 1.32 (1.26, 1.39) |
| Abbreviations: CI, confidence intervals; CVD, cardiovascular disease; DBP, diastolic blood pressure; mmHg, millimetres of mercury; Ref, reference category; RR, relative risk; SBP, systolic blood pressure. | | | | | | | | | |
| ^a^Models adjusted for age at recruitment, Townsend deprivation index, smoking status, diabetes, antihypertensive medication use, lipid-lowering medication use, body mass index, total cholesterol, and high-density lipoprotein cholesterol. | | | | | | | | | |

Table S9. Sex-specific RRs for BP and CHD.

| **Blood pressure category** | |  | **Women** | | |  | **Men** | | |
| --- | --- | --- | --- | --- | --- | --- | --- | --- | --- |
|  |  |  | **Cases** | **Age-adjusted RR (95%CI)** | **Multivariable-adjusted RR (95% CI)^a^** |  | **Cases** | **Age-adjusted RR (95%CI)** | **Multivariable-adjusted RR (95% CI)^a^** |
| **SBP, mmHg** | |  |  |  |  |  |  |  |  |
|  | <100 |  | 26 | 1.00 (0.66, 1.50) | 1.11 (0.72, 1.72) |  | 24 | 1.93 (1.26, 2.95) | 1.92 (1.19, 3.09) |
|  | 100-<105 |  | 26 | 0.48 (0.32, 0.72) | 0.54 (0.35, 0.83) |  | 37 | 1.46 (1.03, 2.07) | 1.57 (1.07, 2.29) |
|  | 105-<110 |  | 100 | 0.86 (0.67, 1.09) | 0.85 (0.65, 1.11) |  | 80 | 0.94 (0.72, 1.21) | 1.09 (0.82, 1.43) |
|  | 110-<115 (Ref) |  | 196 | 1.00 (Ref) | 1.00 (Ref) |  | 200 | 1.00 (Ref) | 1.00 (Ref) |
|  | 115-<120 |  | 312 | 1.12 (0.94, 1.34) | 1.11 (0.92, 1.35) |  | 452 | 1.18 (1.00, 1.40) | 1.19 (0.99, 1.43) |
|  | 120-<125 |  | 467 | 1.34 (1.13, 1.58) | 1.23 (1.03, 1.48) |  | 756 | 1.23 (1.05, 1.44) | 1.21 (1.02, 1.44) |
|  | 125-<130 |  | 563 | 1.43 (1.22, 1.69) | 1.28 (1.08, 1.53) |  | 1,029 | 1.25 (1.07, 1.45) | 1.21 (1.02, 1.42) |
|  | 130-<135 |  | 659 | 1.60 (1.37, 1.88) | 1.44 (1.21, 1.72) |  | 1,397 | 1.42 (1.23, 1.65) | 1.37 (1.16, 1.61) |
|  | 135-<140 |  | 710 | 1.77 (1.51, 2.08) | 1.51 (1.27, 1.79) |  | 1,564 | 1.50 (1.29, 1.73) | 1.38 (1.17, 1.62) |
|  | 140-<145 |  | 668 | 1.81 (1.54, 2.13) | 1.54 (1.29, 1.84) |  | 1,716 | 1.67 (1.44, 1.94) | 1.54 (1.31, 1.81) |
|  | 145-<155 |  | 650 | 2.02 (1.72, 2.38) | 1.73 (1.45, 2.06) |  | 1,577 | 1.73 (1.49, 2.01) | 1.58 (1.35, 1.87) |
|  | 150-<155 |  | 577 | 2.12 (1.80, 2.49) | 1.85 (1.55, 2.21) |  | 1,384 | 1.80 (1.55, 2.09) | 1.62 (1.38, 1.91) |
|  | 155-<160 |  | 424 | 1.92 (1.62, 2.28) | 1.67 (1.38, 2.01) |  | 1,119 | 1.85 (1.59, 2.15) | 1.66 (1.41, 1.96) |
|  | 160-<165 |  | 388 | 2.33 (1.96, 2.77) | 2.09 (1.73, 2.52) |  | 938 | 2.07 (1.77, 2.41) | 1.82 (1.53, 2.15) |
|  | 165-<170 |  | 299 | 2.48 (2.07, 2.98) | 2.19 (1.80, 2.67) |  | 632 | 1.92 (1.64, 2.25) | 1.69 (1.41, 2.01) |
|  | 170-<175 |  | 232 | 2.63 (2.17, 3.18) | 2.31 (1.88, 2.85) |  | 555 | 2.42 (2.06, 2.85) | 2.16 (1.80, 2.58) |
|  | 175-<180 |  | 148 | 2.41 (1.94, 2.99) | 2.02 (1.59, 2.56) |  | 317 | 2.09 (1.75, 2.49) | 1.84 (1.51, 2.24) |
|  | ≥180 |  | 329 | 3.17 (2.65, 3.79) | 2.73 (2.24, 3.32) |  | 595 | 2.34 (2.00, 2.75) | 2.09 (1.75, 2.49) |
|  | Per 10 mmHg |  | 6,774 | 1.15 (1.14, 1.16) | 1.13 (1.12, 1.15) |  | 14,372 | 1.11 (1.10, 1.12) | 1.09 (1.08, 1.10) |
| **DBP, mmHg** | |  |  |  |  |  |  |  |  |
|  | <60 |  | 63 | 1.23 (0.95, 1.59) | 1.34 (1.01, 1.78) |  | 57 | 1.32 (1.01, 1.72) | 1.52 (1.14, 2.03) |
|  | 60-<65 |  | 134 | 0.79 (0.66, 0.95) | 0.83 (0.68, 1.02) |  | 160 | 1.00 (0.85, 1.18) | 1.11 (0.93, 1.32) |
|  | 65-<70 |  | 444 | 0.99 (0.88, 1.11) | 1.05 (0.92, 1.19) |  | 491 | 0.93 (0.84, 1.03) | 0.97 (0.87, 1.09) |
|  | 70-<75 (Ref) |  | 822 | 1.00 (Ref) | 1.00 (Ref) |  | 1,218 | 1.00 (Ref) | 1.00 (Ref) |
|  | 75-<80 |  | 1,251 | 1.16 (1.06, 1.26) | 1.12 (1.02, 1.23) |  | 2,129 | 1.05 (0.97, 1.12) | 1.04 (0.96, 1.12) |
|  | 80-<85 |  | 1,339 | 1.21 (1.11, 1.32) | 1.12 (1.02, 1.24) |  | 2,654 | 1.05 (0.98, 1.13) | 0.99 (0.92, 1.07) |
|  | 85-<90 |  | 1,120 | 1.29 (1.18, 1.41) | 1.14 (1.03, 1.26) |  | 2,733 | 1.14 (1.07, 1.22) | 1.04 (0.97, 1.12) |
|  | 90-<95 |  | 805 | 1.42 (1.29, 1.57) | 1.26 (1.13, 1.40) |  | 2,285 | 1.29 (1.21, 1.39) | 1.16 (1.08, 1.25) |
|  | 95-<100 |  | 437 | 1.54 (1.37, 1.73) | 1.36 (1.20, 1.54) |  | 1,424 | 1.40 (1.30, 1.51) | 1.23 (1.13, 1.34) |
|  | ≥100 |  | 359 | 1.86 (1.65, 2.11) | 1.58 (1.38, 1.82) |  | 1,221 | 1.55 (1.44, 1.68) | 1.32 (1.21, 1.44) |
|  | Per 5 mmHg |  | 6,774 | 1.09 (1.07, 1.10) | 1.06 (1.04, 1.07) |  | 14,372 | 1.07 (1.06, 1.08) | 1.04 (1.03, 1.05) |
| **Hypertension** | |  |  |  |  |  |  |  |  |
|  | No hypertension |  | 1,280 | 1.00 (Ref) | 1.00 (Ref) |  | 1,805 | 1.00 (Ref) | 1.00 (Ref) |
|  | Hypertension |  | 5,494 | 1.66 (1.56, 1.77) | 1.47 (1.37, 1.57) |  | 12,567 | 1.48 (1.40, 1.55) | 1.31 (1.24, 1.39) |
| Abbreviations: CHD, coronary heart disease; CI, confidence intervals; DBP, diastolic blood pressure; mmHg, millimetres of mercury; Ref, reference category; RR, relative risk; SBP, systolic blood pressure. | | | | | | | | | |
| ^a^Models adjusted for age at recruitment, Townsend deprivation index, smoking status, diabetes, antihypertensive medication use, lipid-lowering medication use, body mass index, total cholesterol, and high-density lipoprotein cholesterol. | | | | | | | | | |

Table S10. Sex-specific RRs for BP and stroke.

| **Blood pressure category** | |  | **Women** | | |  | **Men** | | |
| --- | --- | --- | --- | --- | --- | --- | --- | --- | --- |
|  |  |  | **Cases** | **Age-adjusted RR (95%CI)** | **Multivariable-adjusted RR (95% CI)^a^** |  | **Cases** | **Age-adjusted RR (95%CI)** | **Multivariable-adjusted RR (95% CI)^a^** |
| **SBP, mmHg** | |  |  |  |  |  |  |  |  |
|  | <100 |  | 15 | 0.91 (0.53, 1.56) | 0.85 (0.47, 1.54) |  | 5 | 1.35 (0.54, 3.37) | 1.10 (0.34, 3.55) |
|  | 100-<105 |  | 40 | 1.18 (0.83, 1.69) | 1.08 (0.72, 1.61) |  | 6 | 0.80 (0.34, 1.85) | 1.09 (0.46, 2.57) |
|  | 105-<110 |  | 61 | 0.83 (0.61, 1.12) | 0.86 (0.62, 1.19) |  | 39 | 1.58 (1.06, 2.38) | 1.80 (1.13, 2.87) |
|  | 110-<115 (Ref) |  | 125 | 1.00 (Ref) | 1.00 (Ref) |  | 58 | 1.00 (Ref) | 1.00 (Ref) |
|  | 115-<120 |  | 162 | 0.90 (0.71, 1.13) | 0.84 (0.65, 1.08) |  | 139 | 1.25 (0.92, 1.70) | 1.51 (1.06, 2.15) |
|  | 120-<125 |  | 250 | 1.09 (0.88, 1.35) | 1.02 (0.81, 1.28) |  | 223 | 1.24 (0.93, 1.65) | 1.48 (1.05, 2.07) |
|  | 125-<130 |  | 310 | 1.18 (0.96, 1.45) | 1.15 (0.92, 1.44) |  | 333 | 1.36 (1.03, 1.80) | 1.71 (1.23, 2.37) |
|  | 130-<135 |  | 384 | 1.37 (1.12, 1.68) | 1.29 (1.04, 1.61) |  | 383 | 1.29 (0.98, 1.70) | 1.50 (1.08, 2.08) |
|  | 135-<140 |  | 361 | 1.30 (1.06, 1.59) | 1.18 (0.95, 1.47) |  | 433 | 1.35 (1.02, 1.77) | 1.61 (1.16, 2.22) |
|  | 140-<145 |  | 371 | 1.42 (1.16, 1.75) | 1.37 (1.10, 1.71) |  | 472 | 1.46 (1.11, 1.91) | 1.73 (1.25, 2.39) |
|  | 145-<155 |  | 349 | 1.52 (1.24, 1.87) | 1.36 (1.09, 1.70) |  | 521 | 1.79 (1.36, 2.35) | 2.04 (1.48, 2.82) |
|  | 150-<155 |  | 309 | 1.56 (1.27, 1.93) | 1.49 (1.19, 1.87) |  | 419 | 1.67 (1.27, 2.20) | 1.91 (1.38, 2.64) |
|  | 155-<160 |  | 278 | 1.72 (1.39, 2.13) | 1.55 (1.23, 1.96) |  | 380 | 1.89 (1.43, 2.49) | 2.17 (1.57, 3.01) |
|  | 160-<165 |  | 205 | 1.67 (1.33, 2.09) | 1.47 (1.15, 1.88) |  | 320 | 2.09 (1.58, 2.77) | 2.36 (1.70, 3.28) |
|  | 165-<170 |  | 160 | 1.79 (1.41, 2.26) | 1.73 (1.35, 2.23) |  | 228 | 2.06 (1.54, 2.74) | 2.36 (1.68, 3.31) |
|  | 170-<175 |  | 139 | 2.10 (1.65, 2.69) | 1.90 (1.46, 2.48) |  | 180 | 2.28 (1.69, 3.07) | 2.69 (1.90, 3.79) |
|  | 175-<180 |  | 94 | 2.04 (1.55, 2.67) | 1.87 (1.39, 2.51) |  | 123 | 2.34 (1.71, 3.19) | 2.76 (1.92, 3.96) |
|  | ≥180 |  | 203 | 2.55 (2.04, 3.20) | 2.27 (1.77, 2.91) |  | 243 | 2.72 (2.04, 3.62) | 3.06 (2.18, 4.29) |
|  | Per 10 mmHg |  | 3,816 | 1.12 (1.11, 1.14) | 1.11 (1.09, 1.13) |  | 4,505 | 1.13 (1.11, 1.15) | 1.12 (1.10, 1.14) |
| **DBP, mmHg** | |  |  |  |  |  |  |  |  |
|  | <60 |  | 31 | 1.07 (0.74, 1.53) | 0.96 (0.63, 1.47) |  | 13 | 0.96 (0.55, 1.68) | 0.85 (0.46, 1.61) |
|  | 60-<65 |  | 87 | 0.91 (0.72, 1.14) | 0.88 (0.68, 1.14) |  | 50 | 1.03 (0.77, 1.39) | 1.09 (0.79, 1.49) |
|  | 65-<70 |  | 239 | 0.95 (0.81, 1.11) | 1.04 (0.88, 1.23) |  | 175 | 1.12 (0.94, 1.34) | 1.14 (0.93, 1.38) |
|  | 70-<75 (Ref) |  | 465 | 1.00 (Ref) | 1.00 (Ref) |  | 362 | 1.00 (Ref) | 1.00 (Ref) |
|  | 75-<80 |  | 688 | 1.12 (1.00, 1.26) | 1.17 (1.03, 1.33) |  | 622 | 1.03 (0.91, 1.18) | 1.02 (0.89, 1.18) |
|  | 80-<85 |  | 732 | 1.17 (1.04, 1.31) | 1.17 (1.03, 1.33) |  | 827 | 1.11 (0.98, 1.26) | 1.13 (0.99, 1.29) |
|  | 85-<90 |  | 633 | 1.28 (1.13, 1.44) | 1.23 (1.08, 1.40) |  | 866 | 1.23 (1.09, 1.39) | 1.24 (1.08, 1.41) |
|  | 90-<95 |  | 460 | 1.44 (1.26, 1.63) | 1.42 (1.23, 1.64) |  | 721 | 1.39 (1.23, 1.58) | 1.38 (1.20, 1.59) |
|  | 95-<100 |  | 242 | 1.51 (1.30, 1.77) | 1.45 (1.22, 1.72) |  | 426 | 1.43 (1.25, 1.65) | 1.37 (1.18, 1.60) |
|  | ≥100 |  | 239 | 2.22 (1.90, 2.59) | 2.10 (1.76, 2.50) |  | 443 | 1.95 (1.69, 2.24) | 1.96 (1.68, 2.28) |
|  | Per 5 mmHg |  | 3,816 | 1.10 (1.08, 1.12) | 1.09 (1.07, 1.11) |  | 4,505 | 1.10 (1.08, 1.11) | 1.09 (1.08, 1.11) |
| **Hypertension** | |  |  |  |  |  |  |  |  |
|  | No hypertension |  | 730 | 1.00 (Ref) | 1.00 (Ref) |  | 569 | 1.00 (Ref) | 1.00 (Ref) |
|  | Hypertension |  | 3,086 | 1.53 (1.41, 1.67) | 1.44 (1.32, 1.58) |  | 3,936 | 1.36 (1.25, 1.49) | 1.31 (1.19, 1.45) |
| Abbreviations: CI, confidence intervals; DBP, diastolic blood pressure; mmHg, millimetres of mercury; Ref, reference category; RR, relative risk; SBP, systolic blood pressure. | | | | | | | | | |
| ^a^Models adjusted for age at recruitment, Townsend deprivation index, smoking status, diabetes, antihypertensive medication use, lipid-lowering medication use, body mass index, total cholesterol, and high-density lipoprotein cholesterol. | | | | | | | | | |

Table S11. Sex-combined RRs for BP and CVD.

| **Blood pressure category** | |  | **Women** | | |  | **Men** | | |
| --- | --- | --- | --- | --- | --- | --- | --- | --- | --- |
|  |  |  | **Cases** | **Age-adjusted RR (95%CI)** | **Multivariable-adjusted RR (95% CI)^a^** |  | **Cases** | **Age-adjusted RR (95%CI)** | **Multivariable-adjusted RR (95% CI)^a^** |
| **SBP, mmHg** | |  |  |  |  |  |  |  |  |
|  | <100 |  | 39 | 0.37 (0.26, 0.52) | 0.53 (0.36, 0.76) |  | 28 | 1.78 (1.20, 2.62) | 1.76 (1.13, 2.74) |
|  | 100-<105 |  | 66 | 0.30 (0.23, 0.39) | 0.41 (0.30, 0.55) |  | 42 | 1.31 (0.94, 1.82) | 1.46 (1.03, 2.07) |
|  | 105-<110 |  | 157 | 0.34 (0.27, 0.41) | 0.46 (0.37, 0.57) |  | 116 | 1.08 (0.87, 1.35) | 1.22 (0.96, 1.56) |
|  | 110-<115 (Ref) |  | 316 | 0.41 (0.34, 0.48) | 0.54 (0.45, 0.65) |  | 252 | 1.00 (Ref) | 1.00 (Ref) |
|  | 115-<120 |  | 468 | 0.43 (0.37, 0.50) | 0.54 (0.46, 0.65) |  | 576 | 1.20 (1.04, 1.39) | 1.26 (1.07, 1.49) |
|  | 120-<125 |  | 702 | 0.52 (0.45, 0.60) | 0.62 (0.53, 0.73) |  | 953 | 1.23 (1.07, 1.41) | 1.28 (1.09, 1.49) |
|  | 125-<130 |  | 854 | 0.56 (0.49, 0.65) | 0.67 (0.57, 0.79) |  | 1,331 | 1.27 (1.11, 1.45) | 1.32 (1.14, 1.54) |
|  | 130-<135 |  | 1,016 | 0.64 (0.56, 0.74) | 0.76 (0.65, 0.89) |  | 1,728 | 1.37 (1.20, 1.57) | 1.41 (1.21, 1.63) |
|  | 135-<140 |  | 1,047 | 0.69 (0.60, 0.79) | 0.77 (0.66, 0.89) |  | 1,958 | 1.46 (1.28, 1.66) | 1.45 (1.25, 1.68) |
|  | 140-<145 |  | 1,015 | 0.72 (0.63, 0.83) | 0.82 (0.70, 0.96) |  | 2,129 | 1.60 (1.40, 1.82) | 1.60 (1.38, 1.85) |
|  | 145-<155 |  | 965 | 0.79 (0.69, 0.91) | 0.88 (0.75, 1.03) |  | 2,040 | 1.72 (1.51, 1.96) | 1.69 (1.46, 1.96) |
|  | 150-<155 |  | 872 | 0.85 (0.74, 0.98) | 0.97 (0.83, 1.14) |  | 1,748 | 1.73 (1.51, 1.97) | 1.69 (1.45, 1.96) |
|  | 155-<160 |  | 686 | 0.82 (0.71, 0.95) | 0.92 (0.78, 1.08) |  | 1,441 | 1.80 (1.57, 2.06) | 1.75 (1.51, 2.04) |
|  | 160-<165 |  | 578 | 0.92 (0.80, 1.07) | 1.04 (0.88, 1.23) |  | 1,202 | 2.00 (1.74, 2.29) | 1.92 (1.65, 2.23) |
|  | 165-<170 |  | 452 | 1.00 (0.86, 1.17) | 1.15 (0.97, 1.37) |  | 834 | 1.91 (1.66, 2.20) | 1.83 (1.56, 2.14) |
|  | 170-<175 |  | 357 | 1.08 (0.92, 1.27) | 1.21 (1.01, 1.44) |  | 701 | 2.30 (1.99, 2.66) | 2.25 (1.91, 2.64) |
|  | 175-<180 |  | 231 | 1.00 (0.84, 1.20) | 1.09 (0.89, 1.33) |  | 422 | 2.08 (1.78, 2.43) | 2.02 (1.70, 2.41) |
|  | ≥180 |  | 510 | 1.32 (1.13, 1.53) | 1.43 (1.20, 1.69) |  | 796 | 2.35 (2.04, 2.71) | 2.28 (1.95, 2.67) |
|  | Per 10 mmHg |  | 10,331 | 1.14 (1.13, 1.15) | 1.12 (1.11, 1.14) |  | 18,297 | 1.12 (1.11, 1.13) | 1.10 (1.09, 1.11) |
| **DBP, mmHg** | |  |  |  |  |  |  |  |  |
|  | <60 |  | 90 | 0.48 (0.39, 0.59) | 0.60 (0.47, 0.76) |  | 66 | 1.21 (0.94, 1.54) | 1.29 (0.99, 1.69) |
|  | 60-<65 |  | 216 | 0.35 (0.30, 0.40) | 0.43 (0.36, 0.50) |  | 207 | 1.04 (0.90, 1.20) | 1.12 (0.95, 1.30) |
|  | 65-<70 |  | 667 | 0.41 (0.37, 0.44) | 0.51 (0.46, 0.57) |  | 647 | 0.98 (0.90, 1.08) | 1.02 (0.92, 1.13) |
|  | 70-<75 (Ref) |  | 1,249 | 0.42 (0.39, 0.45) | 0.49 (0.45, 0.53) |  | 1,520 | 1.00 (Ref) | 1.00 (Ref) |
|  | 75-<80 |  | 1,891 | 0.48 (0.45, 0.51) | 0.55 (0.51, 0.60) |  | 2,666 | 1.05 (0.99, 1.12) | 1.05 (0.98, 1.12) |
|  | 80-<85 |  | 2,032 | 0.51 (0.47, 0.54) | 0.56 (0.52, 0.60) |  | 3,399 | 1.08 (1.02, 1.15) | 1.05 (0.98, 1.12) |
|  | 85-<90 |  | 1,708 | 0.54 (0.51, 0.58) | 0.57 (0.53, 0.61) |  | 3,486 | 1.17 (1.10, 1.25) | 1.11 (1.04, 1.18) |
|  | 90-<95 |  | 1,234 | 0.61 (0.56, 0.65) | 0.63 (0.58, 0.69) |  | 2,904 | 1.33 (1.25, 1.42) | 1.24 (1.16, 1.33) |
|  | 95-<100 |  | 662 | 0.65 (0.59, 0.71) | 0.67 (0.60, 0.74) |  | 1,800 | 1.44 (1.34, 1.54) | 1.31 (1.22, 1.41) |
|  | ≥100 |  | 582 | 0.84 (0.76, 0.92) | 0.83 (0.75, 0.93) |  | 1,602 | 1.68 (1.56, 1.80) | 1.50 (1.39, 1.62) |
|  | Per 5 mmHg |  | 10,331 | 1.09 (1.08, 1.10) | 1.07 (1.06, 1.08) |  | 18,297 | 1.08 (1.07, 1.08) | 1.05 (1.04, 1.06) |
| **Hypertension** | |  |  |  |  |  |  |  |  |
|  | No hypertension |  | 1,969 | 0.39 (0.36, 0.41) | 0.46 (0.43, 0.49) |  | 2,314 | 1.00 (Ref) | 1.00 (Ref) |
|  | Hypertension |  | 8,362 | 0.66 (0.63, 0.69) | 0.69 (0.65, 0.73) |  | 15,983 | 1.42 (1.36, 1.49) | 1.31 (1.25, 1.38) |
| Abbreviations: CI, confidence intervals; CVD, cardiovascular disease; DBP, diastolic blood pressure; mmHg, millimetres of mercury; Ref, reference category; RR, relative risk; SBP, systolic blood pressure. | | | | | | | | | |
| ^a^Models adjusted for age at recruitment, Townsend deprivation index, smoking status, diabetes, antihypertensive medication use, lipid-lowering medication use, body mass index, total cholesterol, and high-density lipoprotein cholesterol. | | | | | | | | | |

Table S12. Sex-combined RRs for BP and CHD.

| **Blood pressure category** | |  | **Women** | | |  | **Men** | | |
| --- | --- | --- | --- | --- | --- | --- | --- | --- | --- |
|  |  |  | **Cases** | **Age-adjusted RR (95%CI)** | **Multivariable-adjusted RR (95% CI)^a^** |  | **Cases** | **Age-adjusted RR (95%CI)** | **Multivariable-adjusted RR (95% CI)^a^** |
| **SBP, mmHg** | |  |  |  |  |  |  |  |  |
|  | <100 |  | 26 | 0.31 (0.20, 0.46) | 0.47 (0.30, 0.73) |  | 24 | 1.92 (1.26, 2.93) | 1.88 (1.17, 3.02) |
|  | 100-<105 |  | 26 | 0.15 (0.10, 0.22) | 0.23 (0.15, 0.35) |  | 37 | 1.46 (1.03, 2.07) | 1.55 (1.06, 2.26) |
|  | 105-<110 |  | 100 | 0.27 (0.21, 0.34) | 0.36 (0.27, 0.47) |  | 80 | 0.94 (0.72, 1.21) | 1.08 (0.82, 1.43) |
|  | 110-<115 (Ref) |  | 196 | 0.32 (0.26, 0.38) | 0.42 (0.34, 0.52) |  | 200 | 1.00 (Ref) | 1.00 (Ref) |
|  | 115-<120 |  | 312 | 0.36 (0.30, 0.43) | 0.47 (0.38, 0.57) |  | 452 | 1.18 (1.00, 1.40) | 1.20 (1.00, 1.44) |
|  | 120-<125 |  | 467 | 0.43 (0.37, 0.51) | 0.52 (0.43, 0.62) |  | 756 | 1.23 (1.05, 1.43) | 1.22 (1.03, 1.45) |
|  | 125-<130 |  | 563 | 0.47 (0.40, 0.55) | 0.54 (0.46, 0.65) |  | 1,029 | 1.24 (1.07, 1.44) | 1.21 (1.03, 1.43) |
|  | 130-<135 |  | 659 | 0.53 (0.45, 0.62) | 0.61 (0.52, 0.73) |  | 1,397 | 1.41 (1.22, 1.64) | 1.37 (1.17, 1.62) |
|  | 135-<140 |  | 710 | 0.59 (0.51, 0.70) | 0.65 (0.54, 0.77) |  | 1,564 | 1.48 (1.28, 1.71) | 1.38 (1.17, 1.63) |
|  | 140-<145 |  | 668 | 0.61 (0.52, 0.72) | 0.66 (0.56, 0.79) |  | 1,716 | 1.64 (1.42, 1.90) | 1.54 (1.31, 1.81) |
|  | 145-<155 |  | 650 | 0.69 (0.59, 0.81) | 0.75 (0.62, 0.89) |  | 1,577 | 1.70 (1.46, 1.97) | 1.58 (1.35, 1.86) |
|  | 150-<155 |  | 577 | 0.73 (0.62, 0.85) | 0.80 (0.67, 0.96) |  | 1,384 | 1.76 (1.51, 2.04) | 1.62 (1.37, 1.91) |
|  | 155-<160 |  | 424 | 0.66 (0.56, 0.78) | 0.72 (0.60, 0.87) |  | 1,119 | 1.80 (1.55, 2.09) | 1.66 (1.40, 1.96) |
|  | 160-<165 |  | 388 | 0.81 (0.68, 0.96) | 0.91 (0.75, 1.09) |  | 938 | 2.01 (1.72, 2.34) | 1.81 (1.53, 2.14) |
|  | 165-<170 |  | 299 | 0.86 (0.72, 1.03) | 0.95 (0.78, 1.16) |  | 632 | 1.87 (1.59, 2.19) | 1.68 (1.41, 2.00) |
|  | 170-<175 |  | 232 | 0.92 (0.76, 1.11) | 1.00 (0.81, 1.23) |  | 555 | 2.35 (2.00, 2.76) | 2.15 (1.80, 2.57) |
|  | 175-<180 |  | 148 | 0.84 (0.68, 1.04) | 0.87 (0.69, 1.10) |  | 317 | 2.02 (1.69, 2.41) | 1.83 (1.51, 2.23) |
|  | ≥180 |  | 329 | 1.11 (0.93, 1.32) | 1.17 (0.97, 1.43) |  | 595 | 2.26 (1.93, 2.66) | 2.08 (1.74, 2.48) |
|  | Per 10 mmHg |  | 6,774 | 1.15 (1.14, 1.16) | 1.13 (1.12, 1.15) |  | 14,372 | 1.11 (1.10, 1.12) | 1.09 (1.08, 1.10) |
| **DBP, mmHg** | |  |  |  |  |  |  |  |  |
|  | <60 |  | 63 | 0.42 (0.32, 0.54) | 0.57 (0.43, 0.75) |  | 57 | 1.31 (1.00, 1.70) | 1.48 (1.11, 1.98) |
|  | 60-<65 |  | 134 | 0.27 (0.22, 0.32) | 0.35 (0.29, 0.43) |  | 160 | 1.00 (0.85, 1.18) | 1.09 (0.92, 1.31) |
|  | 65-<70 |  | 444 | 0.33 (0.30, 0.37) | 0.44 (0.39, 0.49) |  | 491 | 0.93 (0.84, 1.03) | 0.97 (0.86, 1.09) |
|  | 70-<75 (Ref) |  | 822 | 0.34 (0.31, 0.37) | 0.42 (0.38, 0.46) |  | 1,218 | 1.00 (Ref) | 1.00 (Ref) |
|  | 75-<80 |  | 1,251 | 0.40 (0.37, 0.43) | 0.46 (0.42, 0.50) |  | 2,129 | 1.05 (0.97, 1.12) | 1.04 (0.96, 1.12) |
|  | 80-<85 |  | 1,339 | 0.42 (0.39, 0.45) | 0.47 (0.43, 0.51) |  | 2,654 | 1.05 (0.98, 1.13) | 1.00 (0.93, 1.07) |
|  | 85-<90 |  | 1,120 | 0.44 (0.41, 0.48) | 0.47 (0.43, 0.51) |  | 2,733 | 1.14 (1.06, 1.22) | 1.05 (0.98, 1.13) |
|  | 90-<95 |  | 805 | 0.49 (0.45, 0.54) | 0.51 (0.46, 0.57) |  | 2,285 | 1.29 (1.21, 1.39) | 1.17 (1.09, 1.27) |
|  | 95-<100 |  | 437 | 0.53 (0.47, 0.59) | 0.55 (0.49, 0.62) |  | 1,424 | 1.40 (1.30, 1.51) | 1.25 (1.15, 1.36) |
|  | ≥100 |  | 359 | 0.64 (0.56, 0.71) | 0.63 (0.55, 0.72) |  | 1,221 | 1.56 (1.44, 1.69) | 1.34 (1.23, 1.46) |
|  | Per 5 mmHg |  | 6,774 | 1.09 (1.07, 1.10) | 1.06 (1.04, 1.07) |  | 14,372 | 1.07 (1.06, 1.08) | 1.04 (1.03, 1.05) |
| **Hypertension** | |  |  |  |  |  |  |  |  |
|  | No hypertension |  | 1,280 | 0.32 (0.30, 0.35) | 0.40 (0.37, 0.43) |  | 1,805 | 1.00 (Ref) | 1.00 (Ref) |
|  | Hypertension |  | 5,494 | 0.56 (0.54, 0.60) | 0.59 (0.56, 0.63) |  | 12,567 | 1.45 (1.38, 1.53) | 1.31 (1.24, 1.38) |
| Abbreviations: CI, confidence intervals; CHD, coronary heart disease; DBP, diastolic blood pressure; mmHg, millimetres of mercury; Ref, reference category; RR, relative risk; SBP, systolic blood pressure. | | | | | | | | | |
| ^a^Models adjusted for age at recruitment, Townsend deprivation index, smoking status, diabetes, antihypertensive medication use, lipid-lowering medication use, body mass index, total cholesterol, and high-density lipoprotein cholesterol. | | | | | | | | | |

Table S13. Sex-combined RRs for BP and stroke.

| **Blood pressure category** | |  | **Women** | | |  | **Men** | | |
| --- | --- | --- | --- | --- | --- | --- | --- | --- | --- |
|  |  |  | **Cases** | **Age-adjusted RR (95%CI)** | **Multivariable-adjusted RR (95% CI)^a^** |  | **Cases** | **Age-adjusted RR (95%CI)** | **Multivariable-adjusted RR (95% CI)^a^** |
| **SBP, mmHg** | |  |  |  |  |  |  |  |  |
|  | <100 |  | 15 | 0.66 (0.37, 1.16) | 0.85 (0.45, 1.62) |  | 5 | 1.34 (0.54, 3.34) | 1.08 (0.33, 3.48) |
|  | 100-<105 |  | 40 | 0.85 (0.57, 1.27) | 1.07 (0.67, 1.71) |  | 6 | 0.80 (0.34, 1.84) | 1.09 (0.46, 2.56) |
|  | 105-<110 |  | 61 | 0.60 (0.42, 0.86) | 0.86 (0.57, 1.29) |  | 39 | 1.58 (1.06, 2.38) | 1.80 (1.13, 2.86) |
|  | 110-<115 (Ref) |  | 125 | 0.73 (0.54, 1.00) | 1.00 (0.70, 1.44) |  | 58 | 1.00 (Ref) | 1.00 (Ref) |
|  | 115-<120 |  | 162 | 0.66 (0.49, 0.89) | 0.85 (0.60, 1.21) |  | 139 | 1.25 (0.92, 1.70) | 1.52 (1.06, 2.16) |
|  | 120-<125 |  | 250 | 0.81 (0.61, 1.08) | 1.04 (0.74, 1.45) |  | 223 | 1.23 (0.92, 1.65) | 1.48 (1.05, 2.07) |
|  | 125-<130 |  | 310 | 0.89 (0.67, 1.17) | 1.18 (0.85, 1.64) |  | 333 | 1.35 (1.02, 1.79) | 1.70 (1.23, 2.36) |
|  | 130-<135 |  | 384 | 1.04 (0.79, 1.37) | 1.34 (0.97, 1.86) |  | 383 | 1.27 (0.97, 1.68) | 1.49 (1.08, 2.06) |
|  | 135-<140 |  | 361 | 0.99 (0.75, 1.30) | 1.23 (0.89, 1.71) |  | 433 | 1.33 (1.01, 1.75) | 1.59 (1.15, 2.20) |
|  | 140-<145 |  | 371 | 1.09 (0.83, 1.44) | 1.44 (1.04, 1.99) |  | 472 | 1.43 (1.09, 1.88) | 1.70 (1.24, 2.35) |
|  | 145-<155 |  | 349 | 1.17 (0.88, 1.54) | 1.43 (1.03, 1.99) |  | 521 | 1.75 (1.33, 2.30) | 2.00 (1.45, 2.76) |
|  | 150-<155 |  | 309 | 1.21 (0.91, 1.60) | 1.58 (1.13, 2.19) |  | 419 | 1.63 (1.23, 2.14) | 1.86 (1.35, 2.58) |
|  | 155-<160 |  | 278 | 1.33 (1.00, 1.77) | 1.65 (1.18, 2.30) |  | 380 | 1.83 (1.39, 2.42) | 2.12 (1.53, 2.93) |
|  | 160-<165 |  | 205 | 1.29 (0.96, 1.73) | 1.56 (1.11, 2.20) |  | 320 | 2.03 (1.53, 2.68) | 2.30 (1.65, 3.19) |
|  | 165-<170 |  | 160 | 1.39 (1.03, 1.87) | 1.84 (1.30, 2.61) |  | 228 | 1.99 (1.49, 2.65) | 2.29 (1.64, 3.21) |
|  | 170-<175 |  | 139 | 1.64 (1.20, 2.23) | 2.02 (1.41, 2.89) |  | 180 | 2.20 (1.64, 2.96) | 2.61 (1.85, 3.69) |
|  | 175-<180 |  | 94 | 1.59 (1.14, 2.20) | 1.98 (1.35, 2.90) |  | 123 | 2.25 (1.65, 3.08) | 2.68 (1.87, 3.84) |
|  | ≥180 |  | 203 | 1.99 (1.49, 2.67) | 2.42 (1.71, 3.41) |  | 243 | 2.62 (1.96, 3.49) | 2.98 (2.13, 4.17) |
|  | Per 10 mmHg |  | 3,816 | 1.12 (1.11, 1.14) | 1.11 (1.09, 1.13) |  | 4,505 | 1.13 (1.11, 1.15) | 1.12 (1.10, 1.14) |
| **DBP, mmHg** | |  |  |  |  |  |  |  |  |
|  | <60 |  | 31 | 0.72 (0.50, 1.05) | 0.72 (0.47, 1.10) |  | 13 | 0.96 (0.55, 1.67) | 0.84 (0.45, 1.58) |
|  | 60-<65 |  | 87 | 0.62 (0.49, 0.78) | 0.65 (0.50, 0.85) |  | 50 | 1.03 (0.77, 1.38) | 1.08 (0.79, 1.48) |
|  | 65-<70 |  | 239 | 0.64 (0.55, 0.76) | 0.77 (0.65, 0.92) |  | 175 | 1.12 (0.94, 1.34) | 1.14 (0.93, 1.38) |
|  | 70-<75 (Ref) |  | 465 | 0.68 (0.59, 0.78) | 0.74 (0.64, 0.86) |  | 362 | 1.00 (Ref) | 1.00 (Ref) |
|  | 75-<80 |  | 688 | 0.77 (0.68, 0.87) | 0.87 (0.75, 1.00) |  | 622 | 1.03 (0.91, 1.18) | 1.02 (0.89, 1.18) |
|  | 80-<85 |  | 732 | 0.80 (0.70, 0.91) | 0.87 (0.76, 1.00) |  | 827 | 1.11 (0.98, 1.26) | 1.13 (0.99, 1.29) |
|  | 85-<90 |  | 633 | 0.88 (0.77, 1.00) | 0.92 (0.79, 1.06) |  | 866 | 1.23 (1.09, 1.39) | 1.23 (1.08, 1.41) |
|  | 90-<95 |  | 460 | 0.99 (0.86, 1.13) | 1.05 (0.90, 1.23) |  | 721 | 1.39 (1.23, 1.58) | 1.38 (1.20, 1.59) |
|  | 95-<100 |  | 242 | 1.04 (0.88, 1.22) | 1.07 (0.89, 1.28) |  | 426 | 1.44 (1.25, 1.65) | 1.38 (1.18, 1.61) |
|  | ≥100 |  | 239 | 1.51 (1.29, 1.78) | 1.54 (1.28, 1.85) |  | 443 | 1.96 (1.70, 2.25) | 1.98 (1.70, 2.30) |
|  | Per 5 mmHg |  | 3,816 | 1.10 (1.08, 1.12) | 1.09 (1.07, 1.11) |  | 4,505 | 1.10 (1.08, 1.11) | 1.09 (1.08, 1.11) |
| **Hypertension** | |  |  |  |  |  |  |  |  |
|  | No hypertension |  | 730 | 0.61 (0.54, 0.68) | 0.67 (0.59, 0.76) |  | 569 | 1.00 (Ref) | 1.00 (Ref) |
|  | Hypertension |  | 3,086 | 0.96 (0.88, 1.05) | 0.99 (0.89, 1.10) |  | 3,936 | 1.34 (1.23, 1.46) | 1.30 (1.18, 1.43) |
| Abbreviations: CI, confidence intervals; DBP, diastolic blood pressure; mmHg, millimetres of mercury; Ref, reference category; RR, relative risk; SBP, systolic blood pressure. | | | | | | | | | |
| ^a^Models adjusted for age at recruitment, Townsend deprivation index, smoking status, diabetes, antihypertensive medication use, lipid-lowering medication use, body mass index, total cholesterol, and high-density lipoprotein cholesterol. | | | | | | | | | |

Table S14. Sex-specific RRs for BP and CVD by age group.

| **Blood pressure category** | |  | **Women** | | | | |  | **Men** | | | | |
| --- | --- | --- | --- | --- | --- | --- | --- | --- | --- | --- | --- | --- | --- |
|  |  |  | **Age <55 years**  **(n=95,657)** | |  | **Age ≥55 years**  **(n=139,899)** | |  | **Age <55 years**  **(n=73,106)** | |  | **Age ≥55 years**  **(n=109,987)** | |
|  |  |  | **Cases** | **RR (95%CI)^a^** |  | **Cases** | **RR (95%CI)^a^** |  | **Cases** | **RR (95%CI)^a^** |  | **Cases** | **RR (95%CI)^a^** |
| **SBP, mmHg** | |  |  |  |  |  |  |  |  |  |  |  |  |
|  | <100 |  | 14 | 0.68 (0.40 ,1.16) |  | 25 | 1.13 (0.76 ,1.69) |  | 10 | 2.02 (1.07 ,3.79) |  | 18 | 1.38 (0.86 ,2.21) |
|  | 100-<110 |  | 87 | 0.68 (0.53 ,0.86) |  | 136 | 0.90 (0.74 ,1.08) |  | 54 | 0.99 (0.74 ,1.33) |  | 104 | 1.01 (0.82 ,1.24) |
|  | 110-<120 (Ref) |  | 283 | 1.00 (Ref) |  | 501 | 1.00 (Ref) |  | 286 | 1.00 (Ref) |  | 542 | 1.00 (Ref) |
|  | 120-<130 |  | 420 | 1.29 (1.11 ,1.50) |  | 1136 | 1.24 (1.11 ,1.37) |  | 743 | 1.15 (1.01 ,1.32) |  | 1541 | 1.08 (0.98 ,1.19) |
|  | 130-<140 |  | 393 | 1.59 (1.36 ,1.85) |  | 1670 | 1.47 (1.33 ,1.62) |  | 1070 | 1.45 (1.27 ,1.65) |  | 2616 | 1.17 (1.06 ,1.28) |
|  | 140-<150 |  | 302 | 2.02 (1.72 ,2.38) |  | 1678 | 1.60 (1.44 ,1.76) |  | 998 | 1.88 (1.65 ,2.15) |  | 3171 | 1.33 (1.21 ,1.46) |
|  | 150-<160 |  | 203 | 2.63 (2.19 ,3.16) |  | 1355 | 1.71 (1.54 ,1.89) |  | 580 | 2.08 (1.81 ,2.40) |  | 2609 | 1.42 (1.30 ,1.56) |
|  | 160-<170 |  | 104 | 2.85 (2.28 ,3.58) |  | 926 | 1.95 (1.75 ,2.18) |  | 292 | 2.39 (2.03 ,2.81) |  | 1744 | 1.60 (1.45 ,1.76) |
|  | 170-<180 |  | 51 | 3.35 (2.48 ,4.52) |  | 537 | 2.12 (1.88 ,2.40) |  | 142 | 2.88 (2.35 ,3.53) |  | 981 | 1.80 (1.62 ,2.00) |
|  | ≥180 |  | 38 | 3.77 (2.68 ,5.29) |  | 472 | 2.66 (2.35 ,3.02) |  | 76 | 2.78 (2.16 ,3.59) |  | 720 | 1.95 (1.74 ,2.18) |
|  | Per 10 mmHg |  | 1,895 | 1.24 (1.21 ,1.27) |  | 8,436 | 1.12 (1.11 ,1.13) |  | 4,251 | 1.18 (1.16 ,1.20) |  | 14,046 | 1.10 (1.09 ,1.11) |
|  |  |  | P-interaction=0.001 | | | | | | | | | | |
| **DBP, mmHg** | |  |  |  |  |  |  |  |  |  |  |  |  |
|  | <60 |  | 11 | 0.66 (0.36 ,1.20) |  | 79 | 1.16 (0.93 ,1.46) |  | 11 | 1.06 (0.59 ,1.92) |  | 55 | 1.20 (0.92 ,1.57) |
|  | 60-<70 |  | 144 | 0.73 (0.60 ,0.87) |  | 739 | 0.90 (0.83 ,0.98) |  | 174 | 0.95 (0.81 ,1.12) |  | 680 | 0.97 (0.90 ,1.06) |
|  | 70-<80 (Ref) |  | 520 | 1.00 (Ref) |  | 2620 | 1.00 (Ref) |  | 825 | 1.00 (Ref) |  | 3361 | 1.00 (Ref) |
|  | 80-<90 |  | 657 | 1.42 (1.27 ,1.60) |  | 3083 | 1.10 (1.04 ,1.16) |  | 1538 | 1.34 (1.23 ,1.46) |  | 5347 | 1.03 (0.99 ,1.08) |
|  | 90-<100 |  | 404 | 2.06 (1.81 ,2.34) |  | 1492 | 1.25 (1.17 ,1.33) |  | 1231 | 1.89 (1.73 ,2.07) |  | 3473 | 1.19 (1.14 ,1.25) |
|  | ≥100 |  | 159 | 3.07 (2.57 ,3.67) |  | 423 | 1.63 (1.47 ,1.80) |  | 472 | 2.37 (2.11 ,2.65) |  | 1130 | 1.43 (1.33 ,1.53) |
|  | Per 5 mmHg |  | 1,895 | 1.20 (1.18 ,1.23) |  | 8,436 | 1.07 (1.06 ,1.08) |  | 4,251 | 1.15 (1.13 ,1.17) |  | 14,046 | 1.05 (1.04 ,1.06) |
|  |  |  | P-interaction<0.001 | | | | | | | | | | |
| **Hypertension** | |  |  |  |  |  |  |  |  |  |  |  |  |
|  | No hypertension | | 525 | 1.00 (Ref) |  | 1,444 | 1.00 (Ref) |  | 686 | 1.00 (Ref) |  | 1,628 | 1.00 (Ref) |
|  | Hypertension | | 1,370 | 2.08 (1.88 ,2.31) |  | 6,992 | 1.49 (1.41 ,1.58) |  | 3,565 | 1.74 (1.61 ,1.89) |  | 12,418 | 1.34 (1.27 ,1.41) |
|  |  | | P-interaction=0.007 | | | | | | | | | | |
| Abbreviations: CI, confidence intervals; CVD, cardiovascular disease; DBP, diastolic blood pressure; mmHg, millimetres of mercury; Ref, reference category; RR, relative risk; SBP, systolic blood pressure. | | | | | | | | | | | | | |
| ^a^Models adjusted for age at recruitment. | | | | | | | | | | | | | |

Table S15. Sex-specific RRs for BP and CVD by menopausal status among women participants only.

| **Blood pressure category** | |  | **Pre-menopause**  **(n=47,837)** | |  | **Menopause**  **(n=166,328)** | |
| --- | --- | --- | --- | --- | --- | --- | --- |
|  |  |  | **Cases** | **RR (95%CI)^a^** |  | **Cases** | **RR (95%CI)^a^** |
| **SBP, mmHg** | |  |  |  |  |  |  |
|  | <100 |  | 5 | 0.52 (0.21 ,1.28) |  | 33 | 1.12 (0.79 ,1.59) |
|  | 100-<110 |  | 35 | 0.60 (0.41 ,0.88) |  | 171 | 0.87 (0.73 ,1.03) |
|  | 110-<120 (Ref) |  | 119 | 1.00 (Ref) |  | 604 | 1.00 (Ref) |
|  | 120-<130 |  | 161 | 1.35 (1.07 ,1.71) |  | 1,293 | 1.23 (1.12 ,1.36) |
|  | 130-<140 |  | 125 | 1.63 (1.27 ,2.09) |  | 1,813 | 1.46 (1.33 ,1.60) |
|  | 140-<150 |  | 89 | 2.27 (1.72 ,2.99) |  | 1,801 | 1.61 (1.47 ,1.77) |
|  | 150-<160 |  | 44 | 2.39 (1.69 ,3.38) |  | 1,435 | 1.74 (1.58 ,1.91) |
|  | 160-<170 |  | 35 | 4.40 (3.02 ,6.43) |  | 967 | 1.98 (1.79 ,2.19) |
|  | 170-<180 |  | 12 | 3.89 (2.15 ,7.06) |  | 559 | 2.17 (1.93 ,2.44) |
|  | ≥180 |  | 8 | 4.56 (2.23 ,9.35) |  | 491 | 2.72 (2.41 ,3.07) |
|  | Per 10 mmHg |  | 633 | 1.30 (1.24 ,1.35) |  | 9,167 | 1.13 (1.12 ,1.14) |
|  |  |  | P-interaction<0.001 | | | | |
| **DBP, mmHg** | |  |  |  |  |  |  |
|  | <60 |  | 5 | 0.79 (0.32 ,1.92) |  | 84 | 1.14 (0.92 ,1.42) |
|  | 60-<70 |  | 45 | 0.59 (0.43 ,0.82) |  | 801 | 0.90 (0.83 ,0.97) |
|  | 70-<80 (Ref) |  | 175 | 1.00 (Ref) |  | 2,828 | 1.00 (Ref) |
|  | 80-<90 |  | 231 | 1.77 (1.46 ,2.16) |  | 3,335 | 1.11 (1.05 ,1.16) |
|  | 90-<100 |  | 128 | 2.52 (2.00 ,3.16) |  | 1,638 | 1.27 (1.20 ,1.35) |
|  | ≥100 |  | 49 | 3.81 (2.78 ,5.23) |  | 481 | 1.69 (1.54 ,1.87) |
|  | Per 5 mmHg |  | 633 | 1.26 (1.22 ,1.30) |  | 9,167 | 1.07 (1.06 ,1.08) |
|  |  |  | P-interaction<0.001 | | | | |
| **Hypertension** | |  |  |  |  |  |  |
|  | No hypertension | | 199 | 1.00 (Ref) |  | 1,650 | 1.00 (Ref) |
|  | Hypertension | | 1,434 | 2.39 (2.02 ,2.83) |  | 7,517 | 1.52 (1.44 ,1.60) |
|  |  | | P-interaction=0.009 | | | | |
| Abbreviations: CI, confidence intervals; CVD, cardiovascular disease; DBP, diastolic blood pressure; mmHg, millimetres of mercury; Ref, reference category; RR, relative risk; SBP, systolic blood pressure. | | | | | | | |
| ^a^Models adjusted for age at recruitment. | | | | | | | |

Table S16. Sex-specific RRs for BP and CVD in sensitivity analyses restricting to participants not taking antihypertensive medication at baseline.

| **Blood pressure category** | |  | **Women**  **(n=188,291)** | |  | **Men**  **(n=137,059)** | |
| --- | --- | --- | --- | --- | --- | --- | --- |
|  |  |  | **Cases** | **RR (95%CI)^a^** |  | **Cases** | **RR (95%CI)^a^** |
| **SBP, mmHg** | |  |  |  |  |  |  |
|  | <100 |  | 33 | 0.98 (0.68, 1.40) |  | 13 | 1.31 (0.75, 2.30) |
|  | 100-<105 |  | 53 | 0.75 (0.56, 1.01) |  | 27 | 1.16 (0.77, 1.73) |
|  | 105-<110 |  | 126 | 0.85 (0.69, 1.05) |  | 83 | 1.09 (0.84, 1.42) |
|  | 110-<115 (Ref) |  | 245 | 1.00 (Ref) |  | 177 | 1.00 (Ref) |
|  | 115-<120 |  | 364 | 1.08 (0.92, 1.27) |  | 418 | 1.23 (1.03, 1.47) |
|  | 120-<125 |  | 473 | 1.16 (0.99, 1.35) |  | 674 | 1.28 (1.08, 1.51) |
|  | 125-<130 |  | 568 | 1.30 (1.12, 1.51) |  | 877 | 1.29 (1.09, 1.51) |
|  | 130-<135 |  | 657 | 1.49 (1.29, 1.73) |  | 1,151 | 1.47 (1.25, 1.72) |
|  | 135-<140 |  | 638 | 1.56 (1.34, 1.81) |  | 1,238 | 1.57 (1.34, 1.84) |
|  | 140-<145 |  | 600 | 1.65 (1.42, 1.92) |  | 1,324 | 1.77 (1.51, 2.07) |
|  | 145-<155 |  | 564 | 1.83 (1.57, 2.13) |  | 1,207 | 1.93 (1.65, 2.26) |
|  | 150-<155 |  | 517 | 2.05 (1.76, 2.39) |  | 1,043 | 2.06 (1.76, 2.42) |
|  | 155-<160 |  | 403 | 2.02 (1.72, 2.37) |  | 859 | 2.16 (1.84, 2.55) |
|  | 160-<165 |  | 329 | 2.22 (1.88, 2.62) |  | 703 | 2.39 (2.03, 2.83) |
|  | 165-<170 |  | 264 | 2.41 (2.02, 2.87) |  | 484 | 2.25 (1.89, 2.67) |
|  | 170-<175 |  | 217 | 2.76 (2.29, 3.32) |  | 412 | 2.76 (2.31, 3.29) |
|  | 175-<180 |  | 146 | 2.59 (2.11, 3.19) |  | 245 | 2.42 (2.00, 2.94) |
|  | ≥180 |  | 296 | 3.13 (2.64, 3.72) |  | 502 | 2.88 (2.43, 3.42) |
|  | Per 10 mmHg |  | 6,493 | 1.16 (1.15, 1.18) |  | 11,437 | 1.14 (1.13, 1.16) |
| **DBP, mmHg** | |  |  |  |  |  |  |
|  | <60 |  | 44 | 0.84 (0.62, 1.14) |  | 20 | 0.62 (0.40, 0.97) |
|  | 60-<65 |  | 140 | 0.81 (0.68, 0.97) |  | 121 | 0.99 (0.82, 1.20) |
|  | 65-<70 |  | 426 | 0.94 (0.84, 1.06) |  | 396 | 0.97 (0.86, 1.09) |
|  | 70-<75 (Ref) |  | 796 | 1.00 (Ref) |  | 933 | 1.00 (Ref) |
|  | 75-<80 |  | 1,194 | 1.19 (1.09, 1.30) |  | 1,647 | 1.10 (1.01, 1.19) |
|  | 80-<85 |  | 1,282 | 1.32 (1.21, 1.44) |  | 2,125 | 1.20 (1.11, 1.29) |
|  | 85-<90 |  | 1,061 | 1.45 (1.33, 1.59) |  | 2,180 | 1.35 (1.25, 1.46) |
|  | 90-<95 |  | 749 | 1.61 (1.46, 1.78) |  | 1,800 | 1.55 (1.43, 1.68) |
|  | 95-<100 |  | 435 | 1.88 (1.67, 2.11) |  | 1,129 | 1.69 (1.55, 1.85) |
|  | ≥100 |  | 366 | 2.30 (2.03, 2.60) |  | 1,086 | 2.02 (1.85, 2.20) |
|  | Per 5 mmHg |  | 6,493 | 1.13 (1.11, 1.14) |  | 11,437 | 1.11 (1.10, 1.12) |
| **Hypertension** | |  |  |  |  |  |  |
|  | No hypertension | | 1,424 | 1.00 (Ref) |  | 1,613 | 1.00 (Ref) |
|  | Hypertension | | 5,069 | 1.69 (1.59, 1.80) |  | 9,824 | 1.57 (1.49, 1.65) |
| Abbreviations: CI, confidence intervals; CVD, cardiovascular disease; DBP, diastolic blood pressure; mmHg, millimetres of mercury; Ref, reference category; RR, relative risk; SBP, systolic blood pressure. | | | | | | | |
| ^a^Models adjusted for age at recruitment. | | | | | | | |

Table S17. Sex-specific RRs for BP and CVD in sensitivity analyses restricting to participants with ≥2 years of follow-up.

| **Blood pressure category** | |  | **Women**  **(n234,553)** | |  | **Men**  **(n=182,982)** | |
| --- | --- | --- | --- | --- | --- | --- | --- |
|  |  |  | **Cases** | **RR (95%CI)^a^** |  | **Cases** | **RR (95%CI)^a^** |
| **SBP, mmHg** | |  |  |  |  |  |  |
|  | <100 |  | 33 | 0.86 (0.60, 1.23) |  | 24 | 1.71 (1.12, 2.60)* |
|  | 100-<105 |  | 60 | 0.75 (0.57, 0.99)* |  | 32 | 1.11 (0.77, 1.61) |
|  | 105-<110 |  | 133 | 0.77 (0.63, 0.94)* |  | 106 | 1.10 (0.87, 1.38) |
|  | 110-<115 (Ref) |  | 292 | 1.00 (Ref) |  | 227 | 1.00 (Ref) |
|  | 115-<120 |  | 426 | 1.02 (0.88, 1.18) |  | 510 | 1.18 (1.01, 1.38)* |
|  | 120-<125 |  | 649 | 1.24 (1.08, 1.42)* |  | 864 | 1.24 (1.07, 1.44)* |
|  | 125-<130 |  | 787 | 1.33 (1.16, 1.52)* |  | 1,194 | 1.28 (1.11, 1.47)* |
|  | 130-<135 |  | 937 | 1.50 (1.31, 1.71)* |  | 1,565 | 1.41 (1.22, 1.62)* |
|  | 135-<140 |  | 962 | 1.57 (1.38, 1.79)* |  | 1,733 | 1.46 (1.27, 1.68)* |
|  | 140-<145 |  | 929 | 1.64 (1.44, 1.87)* |  | 1,890 | 1.62 (1.41, 1.86)* |
|  | 145-<155 |  | 874 | 1.76 (1.54, 2.01)* |  | 1,801 | 1.75 (1.52, 2.00)* |
|  | 150-<155 |  | 799 | 1.89 (1.65, 2.17)* |  | 1,542 | 1.76 (1.53, 2.03)* |
|  | 155-<160 |  | 636 | 1.86 (1.61, 2.14)* |  | 1,262 | 1.83 (1.59, 2.11)* |
|  | 160-<165 |  | 524 | 2.03 (1.75, 2.34)* |  | 1,063 | 2.06 (1.79, 2.38)* |
|  | 165-<170 |  | 409 | 2.19 (1.88, 2.55)* |  | 739 | 1.98 (1.70, 2.30)* |
|  | 170-<175 |  | 326 | 2.38 (2.03, 2.79)* |  | 621 | 2.40 (2.06, 2.79)* |
|  | 175-<180 |  | 207 | 2.16 (1.81, 2.59)* |  | 371 | 2.16 (1.83, 2.54)* |
|  | ≥180 |  | 447 | 2.77 (2.39, 3.22)* |  | 699 | 2.44 (2.10, 2.83)* |
|  | Per 10 mmHg |  | 9,430 | 1.14 (1.13, 1.15)* |  | 16,243 | 1.12 (1.11, 1.13)* |
| **DBP, mmHg** | |  |  |  |  |  |  |
|  | <60 |  | 82 | 1.15 (0.92, 1.44) |  | 52 | 1.09 (0.82, 1.44)* |
|  | 60-<65 |  | 182 | 0.77 (0.66, 0.90)* |  | 183 | 1.04 (0.89, 1.21) |
|  | 65-<70 |  | 596 | 0.95 (0.86, 1.05)* |  | 571 | 0.98 (0.89, 1.08) |
|  | 70-<75 (Ref) |  | 1,150 | 1.00 (Ref) |  | 1,349 | 1.00 (Ref) |
|  | 75-<80 |  | 1,746 | 1.15 (1.07, 1.24) |  | 2,385 | 1.06 (0.99, 1.13)* |
|  | 80-<85 |  | 1,872 | 1.21 (1.12, 1.30)* |  | 3,026 | 1.09 (1.02, 1.16)* |
|  | 85-<90 |  | 1,565 | 1.29 (1.19, 1.39)* |  | 3,084 | 1.17 (1.10, 1.25)* |
|  | 90-<95 |  | 1,119 | 1.42 (1.31, 1.54)* |  | 2,577 | 1.33 (1.24, 1.42)* |
|  | 95-<100 |  | 606 | 1.53 (1.39, 1.69)* |  | 1,593 | 1.43 (1.33, 1.54)* |
|  | ≥100 |  | 512 | 1.93 (1.74, 2.14)* |  | 1,423 | 1.67 (1.55, 1.79)* |
|  | Per 5 mmHg |  | 9,430 | 1.09 (1.08, 1.10)* |  | 16,243 | 1.07 (1.07, 1.08)* |
| **Hypertension** | |  |  |  |  |  |  |
|  | No hypertension | | 1,799 | 1.00 (Ref) |  | 2,075 | 1.00 (Ref) |
|  | Hypertension | | 7,631 | 1.62 (1.54, 1.70)* |  | 14,168 | 1.45 (1.38, 1.51)* |
| Abbreviations: CI, confidence intervals; CVD, cardiovascular disease; DBP, diastolic blood pressure; mmHg, millimetres of mercury; Ref, reference category; RR, relative risk; SBP, systolic blood pressure. | | | | | | | |
| ^a^Models adjusted for age at recruitment. | | | | | | | |

Table S18. Sex-specific RRs for BP and CVD in sensitivity analyses imputing missing covariate data via Multivariate Imputation by Chained Equations (MICE) over five iterations.

| **Blood pressure category** | | |  | **Women**  **(n=235,556)** | |  | **Men**  **(n=185,093)** | |
| --- | --- | --- | --- | --- | --- | --- | --- | --- |
|  |  |  |  | **Cases** | **RR (95%CI)^a^** |  | **Cases** | **RR (95%CI)^a^** |
| **SBP, mmHg** | | |  |  |  |  |  |  |
|  | | Per 10 mmHg |  | 10,331 | 1.12 (1.11, 1.13) |  | 18,297 | 1.10 (1.09, 1.11) |
| **DBP, mmHg** | | |  |  |  |  |  |  |
|  | Per 5 mmHg | |  | 10,331 | 1.07 (1.06, 1.08) |  | 18,297 | 1.05 (1.04, 1.06) |
| **Hypertension** | | |  |  |  |  |  |  |
|  | No hypertension | | | 1,969 | 1.00 (Ref) |  | 2,314 | 1.00 (Ref) |
|  | Hypertension | | | 8,362 | 1.45 (1.37, 1.52)* |  | 15,983 | 1.30 (1.24, 1.36)* |
| Abbreviations: CI, confidence intervals; CVD, cardiovascular disease; DBP, diastolic blood pressure; mmHg, millimetres of mercury; Ref, reference category; RR, relative risk; SBP, systolic blood pressure. | | | | | | | | |
| ^a^Models adjusted for age at recruitment, Townsend deprivation index, smoking status, diabetes, antihypertensive medication use, lipid-lowering medication use, body mass index, total cholesterol, and high-density lipoprotein cholesterol. | | | | | | | | |

Table S19. Baseline characteristics of 51,375 UK Biobank participants with BP measurements at baseline (2006-2010) and follow-up (2014+) who were included in the subsample for assessment of regression dilution bias.

| **Characteristics** | | | **Overall** |  | **Women** |  | **Men** |
| --- | --- | --- | --- | --- | --- | --- | --- |
|  |  |  | **(n = 51,375)** |  | **(n = 26,839)** |  | **(n = 24,536)** |
| Incident CVD cases during follow-up, n | | | 2,276 |  | 609 |  | 1,667 |
| Age, mean (SD) | | | 54.6 (7.6) |  | 54.1 (7.4) |  | 55.2 (7.7) |
| Ethnicity | | |  |  |  |  |  |
|  | White | | 49,505 (96.7%) |  | 25,875 (96.6%) |  | 23,630 (96.7%) |
|  | Other^a^ | | 1,710 (3.3%) |  | 899 (3.4%) |  | 811 (3.3%) |
| Socioeconomic status | | |  |  |  |  |  |
|  | Townsend deprivation index score, median (IQR) | | -2.6 (-3.9, -0.5) |  | -2.6 (-3.9, -0.4) |  | -2.6 (-3.9, -0.5) |
|  | Townsend deprivation fifths: | |  |  |  |  |  |
|  |  | First (least deprived) | 22,411 (43.6%) |  | 11,549 (43.0%) |  | 10,862 (44.3%) |
|  |  | Second | 10,900 (21.2%) |  | 5,687 (21.2%) |  | 5,213 (21.3%) |
|  |  | Third | 7,509 (14.6%) |  | 4,006 (14.9%) |  | 3,503 (14.3%) |
|  |  | Fourth | 5,856 (11.4%) |  | 3,109 (11.6%) |  | 2,747 (11.2%) |
|  |  | Fifth (most deprived) | 4,699 (9.2%) |  | 2,488 (9.3%) |  | 2,211 (9.0%) |
| Smoking status: | | |  |  |  |  |  |
|  | Never | | 31,026 (60.5%) |  | 17,145 (64.0%) |  | 13,881 (56.7%) |
|  | Previous | | 16,890 (33.0%) |  | 8,168 (30.5%) |  | 8,722 (35.6%) |
|  | Current light (<15 cigarettes/day) | | 978 (1.9%) |  | 571 (2.1%) |  | 407 (1.7%) |
|  | Current medium (15-<30 cigarettes/day) | | 789 (1.5%) |  | 358 (1.3%) |  | 431 (1.8%) |
|  | Current heavy (≥30 cigarettes/day) | | 1,570 (3.1%) |  | 537 (2.0%) |  | 1,033 (4.2%) |
| Diabetes | | | 4,720 (9.2%) |  | 2,350 (8.8%) |  | 2,370 (9.7%) |
| Medication use: | | |  |  |  |  |  |
|  | Antihypertensive medication use | | 8,451 (16.5%) |  | 3,376 (12.6%) |  | 5.072 (20.7%) |
|  | Lipid-lowering medication use | | 4,966 (9.7%) |  | 1,643 (6.1%) |  | 3,323 (13.5%) |
| Blood Pressure (mmHg): | | |  |  |  |  |  |
|  | SBP, mean(SD) | | 135.0 (17.7) |  | 131.4 (18.1) |  | 139.1 (16.4) |
|  | DBP, mean (SD) | | 81.6 (9.9) |  | 79.6 (9.7) |  | 83.9 (9.6) |
|  | Hypertension | | 34,892 (67.9%) |  | 15,838 (59.0%) |  | 19,054 (77.7%) |
| BMI (kg/m^2^): | | |  |  |  |  |  |
|  | BMI, mean (SD) | | 26.6 (4.3) |  | 26.1 (4.6) |  | 27.2 (3.8) |
|  | Overweight (25-<30) | | 22,103 (43.0%) |  | 9,461 (35.3%) |  | 12,642 (51.5%) |
|  | Obesity (≥30) | | 9,417 (18.3) |  | 4,581 (17.1%) |  | 4,836 (19.7%) |
| Serum lipids (mmol/L): | | |  |  |  |  |  |
|  | Total cholesterol, mean (SD) | | 5.8 (1.1) |  | 5.9 (1.1) |  | 5.6 (1.1) |
|  | HDL-C, mean (SD) | | 1.5 (0.4) |  | 1.6 (0.4) |  | 1.3 (0.3) |
| Numbers are participant numbers (%), with % representing the column percentage estimated excluding participants with missing responses, unless otherwise specified. | | | | | | | |
| Abbreviations: BMI, body mass index; CVD, cardiovascular disease; DBP, diastolic blood pressure; HDL-C, high-density lipoprotein cholesterol; IQR, interquartile range; kg/m^2^, kilogram per square meter; mmHg, millimetres of mercury; mmol/L, millimoles per litre; SBP, systolic blood pressure; SD, standard deviation. | | | | | | | |
| ^a^Includes Asian or Asian British, black or black British, Caribbean, African, any other black background, Chinese, other ethnic group, white and black Caribbean, white and black African, white and Asian, any other mixed background, Indian, Pakistani, Bangladeshi, any other Asian background. | | | | | | | |

**References**

1. UK Biobank Coordinating Centre. *Blood Pressure Measurement Manual: Version 1.0.* Oxford: UK Biobank; 2011. Available from: https://biobank.ndph.ox.ac.uk/showcase/ukb/docs/Bloodpressure.pdf

2. UK Data Service. *2011 Census Aggregate Data (England and Wales, Scotland, and Northern Ireland): UK Data Service.* Colchester: UK Data Archive; 2015. Available from: https://dx.doi.org/10.5257/census/aggregate-2011-2
